# Atherogenic index of plasma and coronary artery disease: a systematic review and meta-analysis of observational studies

**DOI:** 10.1186/s12933-025-02582-2

**Published:** 2025-01-22

**Authors:** Ramin Assempoor, Mohammad Shahabaddin Daneshvar, Aryan Taghvaei, Alireza Sattari Abroy, Amir Azimi, John R. Nelson, Kaveh Hosseini

**Affiliations:** 1 Cardiovascular Diseases Research Institute, Tehran Heart Center, Tehran University of Medical Sciences, North Kargar Ave, Tehran, 1995614331 Iran; 2 Rajaie Cardiovascular Medical and Research Center, Iran University of Medical Sciences, Tehran, Iran; 3 California Cardiovascular Institute, Fresno, CA USA; 4 Cardiac Primary Prevention Research Center, Cardiovascular Diseases Research Institute, Tehran University of Medical Sciences, Tehran, Iran

**Keywords:** Atherogenic index of plasma, Coronary artery disease, Prognosis, Severity, Risk, Outcomes

## Abstract

**Background:**

Atherogenic index of plasma (AIP), a novel logarithmic index that combines fasting triglyceride and high-density lipoprotein cholesterol concentrations, is associated with the burden of atherosclerosis. This study aimed to evaluate the relationship between AIP and coronary artery disease (CAD) risk, severity, and prognosis in populations with and without established CAD.

**Methods:**

PubMed, Embase, and Web of Science were systematically searched from the inception of each database to August 13, 2024. Cross-sectional studies, case-control studies, and prospective or retrospective cohort studies using multivariate analysis were included. Given that the true effect size may differ across studies, a random-effects model for all analyses was applied.

**Results:**

Fifty-one observational studies were included in this study. Patients with higher AIP were more likely to have CAD (odds ratio (OR): 2.79, 95% CI 1.75–4.45, *P* < 0.00001). Furthermore, these patients were more likely to have coronary artery calcification (OR: 2.28, 95% CI 1.74–3.00, *P* < 0.00001), multivessel CAD (OR: 2.04, 95% CI 1.50–2.77, *P* < 0.00001), and an increased risk of plaque progression (OR: 1.49, 95% CI 1.17–1.91, *P* = 0.001). In populations without established CAD, higher AIP levels were associated with an increased risk of Major adverse cardiovascular events (MACE) (hazard ratio (HR): 1.28, 95% CI 1.22–1.35, *P* < 0.00001). Interestingly, this finding was consistent in patients presenting with acute coronary syndrome (HR: 1.59, 95% CI 1.33–1.89, *P* < 0.00001) and patients with chronic coronary syndrome or stable CAD (HR: 1.65, 95% CI 1.15–2.37, *P* = 0.007).

**Conclusions:**

This meta-analysis demonstrates that elevated AIP is strongly associated with increased CAD risk, greater severity, and poorer prognosis in populations with and without established CAD. However, more studies are needed to evaluate the predictive performance and determine the optimal cut-off for AIP in different populations.

**Graphical abstract:**

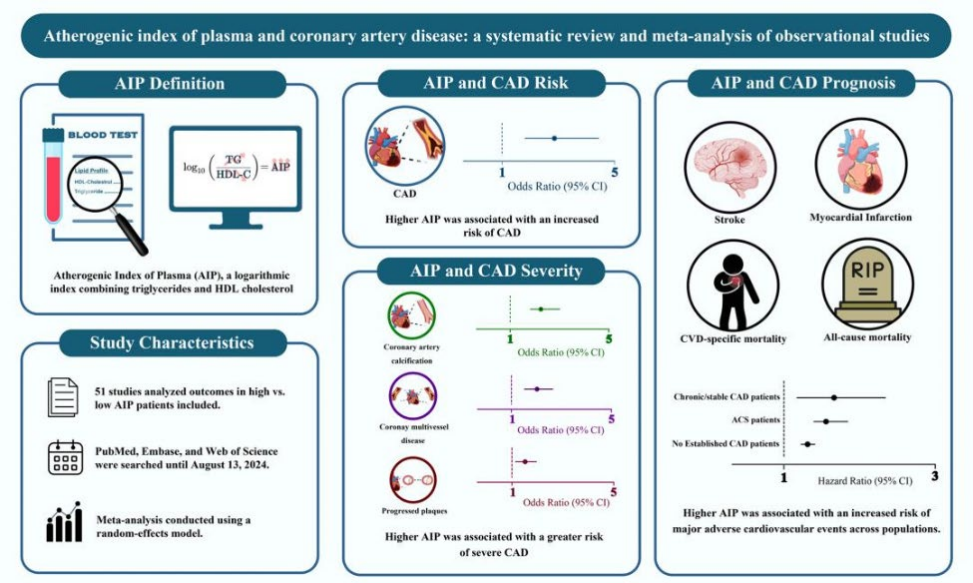

**Supplementary Information:**

The online version contains supplementary material available at 10.1186/s12933-025-02582-2.

## Background

Cardiovascular diseases (CVDs) are among the leading causes of mortality and morbidity worldwide, with coronary artery disease (CAD) comprising a large proportion of these cases [1]. The risk, severity, and prognosis of CAD depend upon multiple factors. In 2001, the atherogenic index of plasma (AIP) was proposed as a potential independent risk factor for CAD due to its correlation with lipoprotein particle size [2]. AIP is calculated as logarithm of [triglyceride (TG) / high-density lipoprotein cholesterol (HDL-C)] and is particularly useful for developing communities where laboratory testing may be inconvenient. AIP has a strong and positive relationship with cholesterol esterification rates, remnant lipoproteinemia, and insulin resistance, all of which contribute to the development of atherosclerosis [3–5]. 

Recent studies have demonstrated that higher AIP levels significantly increase the risk of CAD after adjusting for other risk factors [6]. Moreover, several studies have revealed a significant link between higher AIP levels and worse prognosis in patients with established CAD [7, 8]. Interestingly, similar trends have been observed in patients without CAD and in the general population [9]. To provide more reliable evidence for clinical practice, we conducted a systematic review and meta-analysis to summarize the relationship between AIP, CAD risk, severity, and prognosis in populations with and without established CAD.

## Methods

The authors declare that all essential data are available within the article and the supplementary materials. This systematic review was performed in accordance with the Preferred Reporting Items for Systematic Reviews and Meta-Analyses (PRISMA) reporting guidelines [10]. The study protocol was submitted to the International Prospective Register of Systematic Reviews (https://www.crd.york.ac.uk/prospero/) with the registration number CRD42024610676. This study did not require approval from an institutional review board because we used data from previously published articles only.

### Data sources and searches

We systematically searched the literature by using PubMed, Embase, and Web of Science from their inception up until August 13, 2024. The search strategy was formulated using relevant search terms for: (1) AIP (“Atherogenic index of plasma”) and (2) CVD (“cardiovascular disease,” “coronary artery disease,” “myocardial infarction,” “all-cause mortality,” “cardiovascular death”). A detailed list of the search strategies for all databases is available in supplementary eTable 1. Following the removal of duplicate entries, four investigators (R.A., A.S., A.T., S.D.) screened the remaining articles at the title and abstract level, and then at the full-text and supplementary levels based on the predefined selection criteria. The selected studies were reviewed again for accuracy by the corresponding author (K.H.).

### Study selection

Studies fulfilling all of the predefined inclusion criteria were included: (1) published as full-length articles and written in English; (2) reported the odds ratios (ORs) or hazard ratios (HRs) with 95% confidence interval (CI) for the association between categorized or continuous AIP measurements and the outcomes, after adjusting for potential confounders; (3) specified the increment unit for continuous AIP measurements; (4) reported outcomes related to CAD risk, severity, or prognosis. We excluded abstracts, reviews, nonhuman studies, letters to editors, case reports, and case series. Risk of CAD was determined by the presence of coronary artery stenosis on coronary angiography (CAG). The severity of CAD was assessed by examining coronary artery plaque progression, the presence of multivessel lesions, coronary artery calcification, the no-reflow phenomenon, coronary total occlusion, poor coronary collateral circulation, stent thrombosis, and in-stent restenosis. CAD prognosis was assessed by measuring the incidence of major adverse cardiovascular events (MACE) and their components, including MI, stroke, CVD-specific mortality, and all-cause mortality.

### Data extraction and quality assessment

Four investigators (R.A., A.S., A.T, and S.D.) extracted data under the supervision of the corresponding author (K.H.). We collected the following information on patients’ baseline characteristics and key study details: (1) name of the first author, publication year, country, and study design; (2) participant characteristics, including health status, sample size, mean age, number of male participants, and number of participants with diabetes; (3) methods used for AIP analysis; (4) follow-up duration; (5) reported outcomes; and (6) confounding factors adjusted in the multivariate analyses. We assessed the risk of bias using the Newcastle–Ottawa Scale (NOS), which ranges from 0 (the lowest) to 9 (the highest). Studies with scores of ≥ 6 were considered high-quality studies [11]. 

### Statistical analyses

The Review Manager version 5.4.1 (The Cochrane Collaboration, 2020), and R Project for Statistical Computing were used in all statistical analyses. HRs or ORs, along with their corresponding 95% CIs from the fully adjusted models, were pooled to evaluate the association between AIP and the outcomes. The model with the highest number of variables used for adjustment was chosen for analysis. For studies where AIP was analyzed as a categorical variable, HRs or ORs for the highest versus lowest AIP groups were extracted. In studies where AIP was examined as a continuous variable, HRs or ORs representing the change in outcome per 1-unit increment in AIP were extracted. A *P* < 0.05 was considered as statistical significance for the pooled OR and HR.

Statistical heterogeneity across studies was assessed using the I^2^ statistic, where values of < 25%, 25–50%, and > 50% reflect low, moderate, and high heterogeneity, respectively. Given that the true effect size may differ across studies, we employed a random-effects model for all analyses a priori [12]. For outcomes with I^2^ statistic greater than 50%, we performed subgroup analyses to evaluate the influences of the study characteristics, including gender and diabetes status on the association between AIP and the outcomes. Publication bias was assessed using Egger’s regression asymmetry test, applicable to outcomes with more than nine datasets. Finally, to evaluate the stability of the results, we conducted sensitivity analyses by excluding one individual study at a time.

## Results

### Literature search

Supplementary eFigure 1 shows the process of the database search. Following the aforementioned systematic search, we identified a total of 2247 papers after removing the duplications. Of these, 1977 articles were excluded during the title and abstract screening due to irrelevance. In the next step, 270 papers underwent full-text screening. Of these, 219 articles were excluded for not reporting outcomes of interest and failing to define the increment unit for continuous AIP analyses. Finally, 51 articles were deemed eligible for this meta-analysis. The key findings of the meta-analyses are presented in the Graphical Abstract [13]. We have included the Confirmation of Publication and Licensing Rights for the Graphical Abstract in the Supplementary material.

### Quality assessment

The authors evaluated the quality of the included studies using the NOS, which assigns points to nonrandomized studies based on three criteria: selection, comparability, and outcome. Supplementary eTable 3 provides the details of the NOS scores for each study. In summary, most of the included studies had NOS scores higher than 6, indicating good quality.

### The AIP and CAD risk

**Fig. 1:**
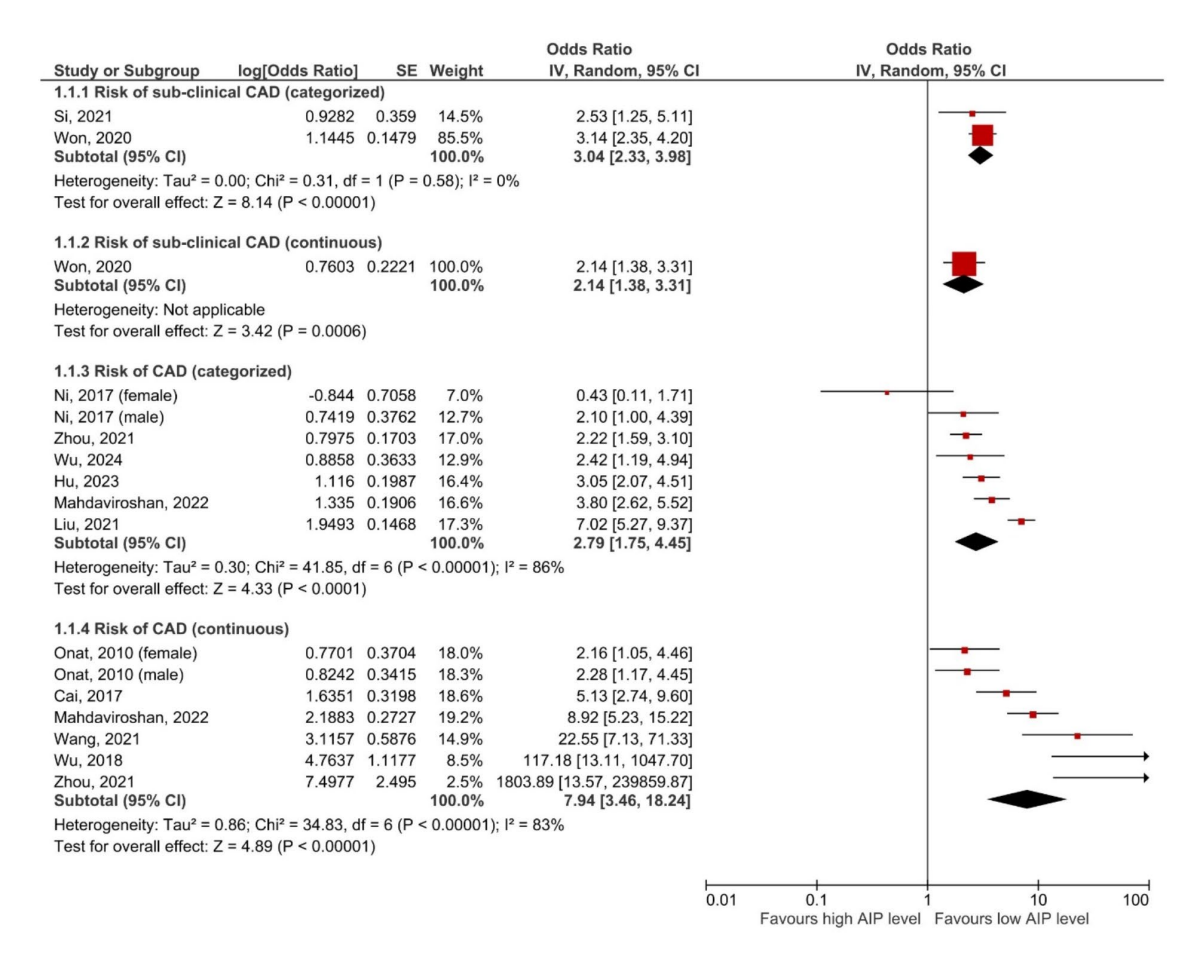
Forest plots showing the pooled result of meta-analysis of CAD risk

Twelve studies assessed the relationship between AIP and CAD risk in asymptomatic patients or those with suspected CAD [6, 14–24]. Details of these studies are presented in Table 1. The pooled results from two studies [14, 15] revealed that asymptomatic patients with higher AIP levels were significantly more likely to have sub-clinical CAD compared to those with lower AIP levels (OR: 3.04, 95% CI 2.33–3.98, I^2^ = 0%, *P* < 0.00001). This finding was consistent when AIP was analyzed as a continuous variable in one study [15] (OR per 1-unit increment of AIP: 2.14, 95% CI 1.38–3.31, *P* = 0.0006). The other ten studies included postmenopausal women [24], patients with type 2 diabetes mellitus [6, 18], and adult patients, with or without symptoms, undergoing CAG for suspected CAD [16, 17, 19–23]. The meta-analysis of six studies [6, 16–20] demonstrated that individuals with higher AIP had a significantly increased risk of CAD (OR: 2.79, 95% CI 1.75–4.45, I^2^ = 86%, *P* < 0.00001, Fig. 1). This association was consistent when AIP was analyzed as a continuous variable in six studies (OR per 1-unit increment of AIP: 7.94, 95% CI 3.46–18.24, I^2^ = 83%, *P* < 0.00001).

**Table 1:** Basic information of the included studies

Study	Design	Country	Participants characteristics	Total, n	Mean age, years	Male, n (%)	Diabetes, n (%)	AIP analysis	AIP index	Endpoint	Variables adjusted in MVA	Follow-up duration
*CAD risk and severity in populations without established CAD*												
Shui et al. [26]	Retrospective cohort	China	Patients who initially underwent CCTA because of suspected CAD, then followed by CCTA again at least half a year apart.	896	62.88 ± 10.63	470 (52.46%)	376 (41.96%)	Categorized	Q1 Q2 Q3 Q4	Coronary artery plaque progress	Age and gender, BMI, smoking status, presence of HTN, DM and obstructive CAD, LDL-C, FPG, baseline GS, and observation time	31.4 months
Wu et al. [17]	Case-control	China	Patients who underwent their first CAG	570	50.2 ± 7.29	484 (84.91%)	128 (22.46%)	Categorized	Q1 (0, 0.04) Q2 (0.05, 0.24) Q3 (0.25, 0.46) Q4 (0.47, 2.14)	1) Symptomatic CAD diagnosed by CAG 2) Multivessel lesion	Age, sex, SBP, BMI, smoking status, drinking status, LDL-C, HbA1c, DM, hypertension, status of antihypertensive drugs, status of antidiabetic drugs, status of antilipidemic drugs, status of antiplatelet drugs	Not reported
Hu et al. [18]	Crosssectional	China	Type2 diabetic patients who underwent coronary angiography	737	61.45 ± 10.03	412 (55.9%)	737 (100%)	Categorized	AIP < 1.17 AIP > 1.17	1) Symptomatic CAD diagnosed by CAG 2) Multivessel lesion	BMI, age, sex, smoking, DBP, SBP, AST, LDL-C, ALB, FIB, D-dimer, FDP, HbA1c, eGFR, Hb, monocytes, and neutrophils	Not reported
Won et al. [28]	Retrospective cohort	South Korea	Asymptomatic patients who underwent at least two CAC evaluations	6927	Not reported	Not reported	Not reported	Continuous	per 0.1-unit increase	Coronary artery plaque progress	Age, sex, and the traditional risk factors of hypertension, diabetes, hyperlipidemia, obesity, and current smoking	39.6 months
Mahdavi-Roshan et al. [19]	Case‒control	Iran	Patients visited the cardiology outpatient clinic due to having clinical signs and/or symptoms of heart disease or for routine check-ups.	3859	51.60 ± 11.86	1624 (42.08%)	490 (13.65%)	Categorized Continuous	Q1 Q2 Q3 Q4 per 1-unit increase	Symptomatic CAD diagnosed by CAG	Age, sex, smoking, family history of heart disease, hypertension, type 2 diabetes mellitus, and statin use	Not reported
Liu et al. [38]	Retrospective cohort	China	Patients with chest pain and suspected acute coronary syndrome who underwent CAG	1131	63.83 ± 11.09	974 (86.11%)	459 (40.58%)	Categorized	AIP < 0.345 AIP > 0.345	Symptomatic CAD diagnosed by CAG	Hypertension, type 2 diabetes mellitus, and smoking	Not reported
Si et al. [14]	Cross-sectional	China	Adults undergoing a health check-up	697	59.6 ± 8.2	333 (47.8%)	121 (17.6%)	Categorized	AIP<-0.253 AIP ≥-0.253	Subclinical CAD diagnosed by CCTA	Age, sex, hypertension, DM, and dyslipidemia	Not reported
Wang et al. [23]	Cross-sectional	China	Adult patients undergoing CAG for suspected CAD	3600	60.4 ± 10.1	2243 (62.3%)	872 (24.2%)	Continuous	per 1-SD increase (0.26)	Symptomatic CAD diagnosed by CAG	Age, sex, smoking, BMI, hypertension, DM, and dyslipidemia	Not reported
Won et al. [27]	Prospective cohort	South Korea	Asymptomatic patients who underwent serial CCTA evaluations	1488	60.9 ± 9.2	876 (58.9%)	353 (23.7%)	Categorized	T1 T2 T3	Coronary artery plaque progress	Age, sex, systolic BP, diastolic BP, BMI, the serum levels of total cholesterol, LDL cholesterol, glucose, the use of aspirin, beta blocker, ACEI/ ARB, statin, and baseline total PAV	Not reported
Zhou et al. [6]	Cross-sectional	China	Adult type 2 diabetes mellitus patients undergoing CAG for suspected CAD	3278	58.96 + 9.94	2473 (75.4%)	3278 (100%)	Categorized Continuous	Q1 (< 0.155) Q2 (0.155, 0.329) Q3 (0.329, 0.491) Q4 (> 0.491) per 1-SD increase (0.25)	Symptomatic CAD diagnosed by CAG	Age, sex, BMI, systolic and diastolic BP, smoking, alcohol drinking, duration of T2DM, hypertension, dyslipidemia, and history of stroke	Not reported
Nam et al. [25]	Retrospective cohort	South Korea	patients who had undergone CAC measurement at least twice by multi-detector computed tomography (CT) at a health check-up center	1124	51.57 ± 7.70	594 (52.85%)	81 (7.20%)	Categorized	T1 T2 T3	Coronary artery plaque progress	Age and sex, BMI, SBP, FPG, LDL-C, exercise, alcohol, smoking, presence of diabetes and hypertension, and baseline ln (CACS + 1)	50.4 months
Won et al. [28]	Cross-sectional	Korea	Adults undergoing a health check-up	6928	52.0 ± 9.7	3977 (57.4%)	776 (11.2%)	Categorized Continuous	Q1 (-0.6, 0.14) Q2 (0.15, 0.35) Q3 (0.36, 0.55) Q4 (0.56, 1.79) per 0.1-unit increase	1) Coronary artery calcification 2) Subclinical CAD diagnosed by CCTA	Age, sex, hypertension, DM, dyslipidemia, obesity, and proteinuria	Not reported
Wu et al. [24]	Case-control	China	Postmenopausal women undergoing CAG for suspected CAD	696	61.7 ± 7.1	0 (0%)	151 (21.7%)	Continuous	per 1-SD increase (0.27)	Symptomatic CAD diagnosed by CAG	Age, hypertension, DM, smoking, heart rate, and FBG	Not reported
Cai et al. [22]	Case-control	China	Adult patients undergoing CAG for suspected CAD	5387	62.2 ± 9.7	3242 (60.2%)	1050 (19.5%)	Continuous	per 1-SD increase (0.31)	Symptomatic CAD diagnosed by CAG	Age, sex, smoking, DM, and hypertension	Not reported
Ni et al. [16]	Cross-sectional	China	Adult patients undergoing CAG for suspected CAD	463	65.3 ± 9.7	306 (66.1%)	83 (17.9%)	Categorized	Q1 Q2 Q3 Q4	Symptomatic CAD diagnosed by CAG	Age, sex, BMI, FBG, homocysteine, and smoking	Not reported
Onat et al. [21]	Prospective cohort	Turkey	Community- derived middle-aged adults	2676	48.9 ± 12.6	1294 (48.4%)	153 (5.7%)	Continuous	per 0.3-unit increase	Symptomatic CAD	Age, serum CRP, systolic BP, smoking status, BMI, total and LDL-cholesterol	7.8 years
*Prognosis in populations without established CAD*												
Liu et al. [32]	Prospective cohort	China	Target subjects invited to a community-based cohort	3820	59.1 ± 8.7	1379 (36.1%)	780 (20.4%)	Categorized continuous	Q1(< -0.2) Q2(-0.2, 0.0) Q3(0.0, 0.2) Q4(≥ 0.2) per 1-unit increase	1) MACE 2) Stroke 3) MI 4) CVD-specific 5) mortality	Age, gender, BMI, current smoking status, cardiovascular disease, hypertension, diabetes mellitus, antihypertensive drugs, antidiabetic drugs, lipid-lowering drugs, LDL-c, and eGFR	7.5 years
Qin et al. [37]	Retrospective cohort	China	Adult patients from NHANES database	18,133	47.56 ± 22.98	8,816 (48.62%)	3,523 (19.43%)	Categorized continuous	Q1 (− 1.25, − 0.29) Q2 (− 0.29, − 0.07) Q3 (− 0.07, 0.15) Q4 (0.15, 1.45) Per 1-SD increase (0.34)	CVD-specific mortality All-cause mortality	Gender, age, race, education, family income poverty ratio, BMI, smoking status, drinking status, low-density lipoprotein cholesterol	Not reported
Qu et al. [42]	Prospective cohort	China	Participants aged 45 years or older without a history of stroke from CHARLS study	8727	58.04 ± 8.75	3985 (46.7%)	1216 (13.9%)	Categorized continuous	Q1 (≤ 0.122) Q2 (0.122, 0.329) Q3 (0.329, 0.562) Q4 (> 0.562) per 1-unit increase	Stroke	Age, gender, marital status, drinking, smoking, residence, SBP, DBP, BMI, hypertension, heart disease, TC, FPG, HbA1c	9 years
Yu et al. [39]	Retrospective cohort	South Korea	Acute decompensated heart failure cases from the JX-ADHF1 study	1248	69.75 ± 9.27	735 (58.90%)	328 (26.28%)	Categorized continuous	Q1 (-0.73 to -0.08) Q2 (-0.08 to 0.08) Q3 (0.08 to 0.24) Q4 (0.24 to 1.55) per 1-unit increase	All-cause mortality	Gender, age, hypertension, diabetes, cerebral infarction and CHD, NYHA classification, LVEF, DBP, NT-proBNP, WBC, RBC, PLT, ALB, AST, GGT, Cr, UA, LDL-C.	3 years
Zhang et al. [43]	Prospective cohort	China	Participants from the Kailuan study, which was an ongoing, prospective, cohort study conducted in the Kailuan community in Tangshan City, China	98,861	51.53 ± 11.59	78,849 (79.76%)	5758 (5.82%)	Categorized continuous	Q1 Q2 Q3 Q4 per 1-unit increase	MI	Age and sex, education, smoking status, drinking status, body mass index, systolic blood pressure, fasting plasma glucose, high sensitivity C-reactive protein, total cholesterol, low-density lipoprotein cholesterol level, history of hypertension, hyperlipidemia, diabetes, antihypertensive drugs, antidiabetic drugs, and lipid-lowering drugs	153.6 months
Zhang et al. [45]	Prospective cohort	China	Participants from the Kailuan study, which was an ongoing, prospective, cohort study conducted in the Kailuan community in Tangshan City, China	97,959	51.40 ± 11.46	78,014 (79.64%)	8981 (9.17%)	Categorized	Q1 ( ≤ − 0.56) Q2 (− 0.56, − 0.16) Q3 (− 0.16, 0.31) Q4 (≥ 0.31)	Stroke	Age and sex, education, smoking status, drinking status, body mass index, physical activity, systolic blood pressure, history of hypertension, hyperlipidemia, diabetes, antihypertensive drugs, antidiabetic drugs, lipid-lowering drugs, fasting plasma glucose, hs-CRP, total cholesterol level, and low-density lipoprotein cholesterol level	12 years
Zhi et al. [33]	Prospective cohort	China	Participants free of a history of MACEs at baseline from the UK Biobank data	361,644	56.19 ± 8.09	161,976 (44.79%)	17,724 (4.90%)	Categorized continuous	Q1 Q2 Q3 Q4 per 1-unit increase	MACE	Age, ethnicity, sex, fasting glucose, low-density lipoprotein cholesterol, serum creatinine, total cholesterol, antihypertensive medication, glucose-lowering medication, history of chronic kidney disease, history of diabetes, history of hypertension, lipid-lowering medication, smoking, and systolic blood pressure	12.19 years
Deng et al. [36]	Prospective cohort	China	Patients who underwent continuous ambulatory peritoneal dialysis	2682	51.3 ± 14.6	1491 (55.6%)	563 (21%)	Categorized continuous	Q1 (< 2.20) Q2 (2.20, 2.97) Q3 (2.97, 4.04) Q4 (≥ 4.04) per 1-unit increase	CVD-specific mortality All-cause mortality	Age, sex, BMI, history of CVD, DM, hemoglobin, platelet, albumin, calcium, phosphate, iPTH, eGFR, hs-CRP, smoking, lipid-lowering medication, antiplatelet medication and Kt/V	35.5 months
Tamosiunas et al. [35]	Prospective cohort	Lithuania	Participants from a survey conducted in Kaunas city	6671	57.64 ± 7.99	3008 (45.09%)	527 (7.90%)	Categorized	quintile 1 quintile 2 quintile 3 quintile 4 quintile 5	CVD-specific mortality All-cause mortality	Age, education, physical activity and smoking status, and biological factors (arterial hypertension, total cholesterol, and fasting glucose)	10 years
Cai et al. [44]	Retrospective cohort	China	Patients with OSA hypertension diagnosed with polysomnography	2281	49.47 ± 10.65	1562 (68.47%)	399 (17.40%)	Categorized continuous	Q1 (< 0.04) Q2 (0.04, 0.22) Q3 (0.22, 0.41) Q4 (≥ 0.41) per 1-SD increase	MI	Age and gender, drinking status, SBP, smoking status, DBP, diabetes, BMI, lipid-lowering drugs, antiplatelet drugs, regularly CPAP treatment, antihypertensive drugs	7.15 years
Hang et al. [31]	Retrospective cohort	China	Patients who had 50 years old, had systolic BP levels of 130–180 mmHg, and had an increased risk of cardiovascular disease.	9323	67.87 ± 9.41	6016 (64.5%)	0 (0%)	Categorized	Q1 (≤ -0.228) Q2 (-0.228, -0.037) Q3 (-0.037, 0.156) Q4 (> 0.156)	MACE	Age, race, treatment arm, body mass index, systolic blood pressure, heart rate, smoking status, serum creatinine, fasting total cholesterol, fasting glucose, previous CVD, previous CKD, aspirin use and statin use	3.22 years
Kim et al. [9]	Prospective cohort	South Korea	Patients from NHIS-HEALS cohort	514,866	58.97 ± 8.70	276,483 (53.7%)	45,321 (8.80%)	Categorized	Q1(<– 0.40) Q2(– 0.40, 0.04) Q3(0.04, 0.50) Q4(≥ 0.50)	MACE CVD-specific mortality	Baseline age, sex, body mass index, smoking, alcohol drinking, physical activities, household income, fasting glucose, systolic blood pressure, low-density lipoprotein cholesterol, and estimated glomerular filtration rate levels.	5 years
Mangalesh et al. [34]	Prospective cohort	India	Apparently healthy patient population with no traditional risk factors for CAD undergoing coronary computed tomography (CT) angiography	366	62 ± 9.63	259 (70.8%)	0 (0%)	Continuous	per 0.1-unit increase	MACE	Sex, age, BMI, WHR, hsCRP, Uric Acid, HbA1c, and Tc/HDLc	2.57 years
Fu et al. [29]	Retrospective cohort	China	Patients with type 2 diabetes mellitus from ACCORD/ACCORDION trial	10,251	62.81 ± 6.65	6299 (61.45%)	10,251 (100%)	Categorized	AIP ≤ 0.34 AIP > 0.34	1) MACE 2) CVD-specific mortality 3) MI 4) Stroke 5) All-cause mortality 6) Congestive heart failure	Age, sex, previous cardiovascular event, smoking, BMI, duration of diabetes, previous congestive heart failure, eGFR, HbA1c, plasma triglycerides, total plasma cholesterol, plasma HDL-C, insulin, biguanide, sulfonylurea, thiazolidinediones, statin, other lipid-lowering medications, niacin, and fibrate	5 years
Hongbing Liu et al. [38]	Prospective cohort	Taiwan	Patients with acute ischemic stroke	1463	60.25 ± 12.31	1022 (69.9%)	331 (22.6%)	Categorized	Q1 (<-0.10) Q2 (-0.10, 0.08) Q3 (0.08, 0.26) Q4 (≥ 0.26)	All-cause mortality	Age, gender, baseline NIHSS, reperfusion therapy, history of lipid-lowering therapy, history of atrial fibrillation, history of diabetes mellitus, glucose, TC, and LDL-C	3 months
Sadeghi et al. [30]	Prospective cohort	Iran	Apparently healthy adults over 35-year-old	6323	50.8 ± 11.7	3068 (48.5%)	686 (10.8%)	Categorized	T1 (< 0.11) T2 (0.11 to 0.21) T3 (> 0.21)	MACE	Age, sex, sociodemographic factors, lifestyle factors (smoking, global dietary index and physical activity), traditional factors (hypertension, diabetes, high LDL, obese, and overweight)	15 years
Ahn et al. [40]	Retrospective cohort	South Korea	Patients diagnosed with antibody-associated vasculitis	167	59.0 ± 16.29	54 (32.3%)	51 (30.5%)	Categorized	AIP < 0.11 AIP ≥ 0.11	Stroke	Gender, BMI, age, and AAV subtypes	3 months
Wang et al. [41]	Cross-sectional	China	Individuals from a large population study (NCRCHS)	11,465	53.93 ± 10.58	5225 (45.57%)	1217 (10.61%)	Categorized continuous	Q1 Q2 Q3 Q4 Per 1 SD (0.32)	Stroke	Age, gender, race, education status, family income, current smoking and drinking status, physical activity, BMI, hypertension, diabetes, family history of stroke, and atrial fibrillation	Not reported
*CAD severity in populations with established CAD*												
Dong et al. [47]	Retrospective cohort	China	CAD patients with chronic total occlusion confirmed by coronary angiography	1093	58.8 ± 10.2	936 (85.6%)	463 (42.4%)	Categorized continuous	Q1 (< 0.01) Q2 (0.01, 0.18) Q3 (0.18, 0.36) Q4 (≥ 0.36) per 1-unit increase	Poor coronary collateral circulation	Age, male, BMI, current smoking, hypertension, T2DM, dyslipidemia, hs-CRP, creatinine, uric acid, FBG, HbA1c, TC, LDL-C, and LVEF, number of lesions and vessels with CTO	4 years
Han et al. [46]	Cross-sectional	China	Patients with CAD undergoing CAG	5238	59.72 ± 10.00	3947 (75.4%)	1884 (36.0%)	Categorized continuous	AIP ≤ 0.15 AIP > 0.15 per 0.01-unit increase	Coronary total occlusion	Male gender, previous CABG, left main disease, ACS, diabetes mellitus, TC, LDL-C, eGFR, age, smoking, and hypertension	Not reported
Abacıoğlu et al. [49]	Retrospective cohort	Turkey	patients with ACS underwent PCI	698	63.3 ± 10.6	483 (69.2%)	59 (8.5%)	Categorized	AIP < 0.32 AIP > 0.32	Stent thrombosis	Not reported	Not reported
Zhu et al. [48]	Retrospective Cohort	China	ACS patients treated with PCI undergoing coronary angiography within 6–18 months	1319	58.40 ± 9.47	1025 (77.7%)	476 (36.1%)	Categorized	Q1 ( ≤ − 0.042) Q2 (− 0.042, 0.136) Q3 (0.136, 0.305) Q4 (> 0.305)	In-stent restenosis	Not reported	11.11 ± 3.04 months
Refaat et al. [50]	Cross-sectional	Egypt	Patients with acute STEMI who underwent primary PCI	400	60.3 ± 11.0	284 (71.0%)	224 (56.0%)	Categorized	AIP ≤ 0.52 AIP > 0.52	No reflow	Not reported	6 months
*Prognosis in patients with ACS*												
Abdu et al. [55]	Retrospective Cohort	China	Patients diagnosed with MINOCA	421	63.95 ± 13.55	220 (52.3%)	83 (19.7%)	Categorized	Q1 (<-0.145) Q2 (-0.145, 0.049) Q3 (0.049, 0.253) Q4 (> 0.253)	MACE	Not reported	34 ± 24.55 months
Won et al. [5]	Retrospective cohort	South Korea	Patients with acute MI presentations from PTRG-DES study underwent successful PCI with DES for obstructive coronary artery disease	3136	64.1 ± 12.4	2192 (69.9%)	942 (30%)	Continuous	per 0.1-unit increase	MACE	Age ≥ 75 years, sex, hypertension, diabetes, dyslipidemia, obesity, smoking, chronic kidney disease, medical therapy, HPR, multivessel disease, bifurcation lesions, CTO lesions, number of stents, total stent length, minimum stent diameter, LVEF	3 years
Kan et al. [54]	Retrospective cohort	China	patients with ACS who underwent either primary or elective PCI	526	62 ± 10	338 (64.3%)	219 (41.6%)	Categorized	T1 (≤ -0.0458) T2 (-0.0458, 0.2262) T3 (> 0.2262)	MACE	Male, age, current smoking, hypertension, diabetes, cardiac failure, NSTE-ACS, CKD, hs-CRP, SYNTAX score, and complete revascularization	Not reported
Liu et al. [56]	Retrospective cohort	China	prediabetic patients with unstable angina pectoris	1096	59.47 ± 9.86	766 (69.9%)	0	Continuous	per 1-unit increase	1) MACE 2) Cardiac death 3) MI	Age, sex (male), BMI, SBP, DBP, smoking, hypertension, dyslipidemia, prior PCI, TC, LDL-C, eGFR, FBG, HbA1c, Cr, CRP, SUA, and medication of statins, ACEI, ARB, CCB, β-blocker, antiplatelet	26.3 ± 6.5 months
Ozen et al. [53]	Retrospective cohort	Turkey	Patients with ACS who underwent urgent coronary angiography	558	59 ± 18	422 (75.8%)	213 (38.2%)	Categorized	AIP ≤ 0.50 AIP > 0.50	MACE	Not reported	1 year
Wang et al. [7]	Retrospective cohort	China	patients with ACS and LDL-C levels below 1.8 mmol/L who underwent PCI	1133	58.6 ± 9.3	966 (85.3%)	485 (42.8%)	Categorized continuous	AIP < 0.11 AIP ≥ 0.11 Per 1-unit increase	1) MACE 2) All-cause death 3) Cardiovascular death 4) MI 5) Stroke 6) revascularization	Sex, age, body mass index (BMI), hypertension, dyslipidemia, diabetes mellitus, previous MI, previous stroke, oral hypoglycemic agents, LDL-C, TC, HbA1c, and uric acid	26 months
Qiao-Yu Shao et al. [52]	Retrospective cohort	China	patients with ACS who underwent primary or elective PCI	1694	60.0 ± 10.4	1296 (76.5%)	776 (45.8%)	Categorized	T1 (< 0.05) T2 (0.05, 0.28) T3 (> 0.28)	MACE	Sex, body mass index, current smoking, hypertension, diabetes mellitus, dyslipidemia, past myocardial infarction, past percutaneous coronary intervention, chronic kidney disease, admission diagnosis with different types of acute coronary syndrome, GRACE risk score, high-sensitivity C-reactive protein, SYNTAX score, complete revascularization, and discharged medications	31 months
Ma et al. [51]	Retrospective cohort	China	Patients with type 2 diabetes and ACS who underwent primary or elective PCI	798	61.3 ± 9.7	580 (72.7%)	798 (100%)	Categorized continuous	Q1 (≤ 0.0147) Q2 (0.0147, 0.1850) Q3 (0.1850, 0.3517) Q4 (> 0.3517) per 1-unit increase	MACE	Age, BMI, hypertension, previous MI, past PCI, peripheral artery disease, cardiac failure, LVEF, serum creatinine, LDL-C, FPG, clinical presentation, CAD severity, lesions > 20 mm long, restenotic lesions, use of drug-coated balloon, complete revascularization, use of insulin	31 months
Anggoro B. Hartopo et al. [57]	Prospective cohort	Indonesia	Hospitalized Patients with Acute MI	277	63.95 ± 9.13	221 (79.8%)	77 (27.8%)	Categorized	AIP < 0.24 AIP ≥ 0.24	All-cause mortality	Not reported	Not reported
*Prognosis in patients with CCS or stable CAD*												
Won et al. [5]	Retrospective Cohort	South Korea	Patients with non-AMI presentations from PTRG-DES study underwent successful PCI with DES for obstructive coronary artery disease	7599	64.5 ± 10.2	5060 (66.6%)	2772 (36.5%)	Continuous	per 0.1-unit increase	MACE	Age ≥ 75 years, sex, hypertension, diabetes, dyslipidemia, obesity, smoking, chronic kidney disease, medical therapy, HPR, multivessel disease, bifurcation lesions, CTO lesions, number of stents, total stent length, minimum stent diameter, LVEF	3 years
Alifu et al. [59]	Retrospective cohort	China	Patients diagnosed with CCS and underwent CAG	404	63.61 ± 9.64	238 (58.9%)	144 (35.6%)	Categorized	Q1 (< -0.064) Q2 (-0.064, 0.130) Q3 (0.130, 0.328) Q4 (> 0.328)	MACE	Age, FBG, LVEF, diabetes, hyperlipidemia, and heart failure	median: 35 months
Zheng et al. [58]	Prospective cohort	China	nondiabetic CAD patients who underwent PCI	5538	57.4 ± 10.1	4394 (79.3%)	0	Categorized	T1 T2 T3	1) MACE 2) All-cause death 3) Cardiac death 4) Revascularization 5) Stroke 6) MI	Age, male, BMI, classification of CAD, hypertension, hyperlipidemia, renal dysfunction, smoker, cerebrovascular diseases, pre-PCI, pre-CABG, pre-myocardial infarction, COPD, peripheral vascular disease, CTO, TVD, creatine, albumin, left main involved, β-blocker at discharge, statin at discharge, ACEI/ARB at discharge, CCB at discharge	28 ± 2.3 months
Qin et al. [8]	Prospective cohort	China	Patients with T2DM who underwent PCI	2356	58.0 ± 9.2	1738 (73.8%)	2356 (100%)	Categorized	AIP < 0.318 AIP > 0.318	1) MACE 2) All-cause death 3) Cardiac death 4) MI 5) revascularization 6) Stroke	Age, body mass index, sex, medical history, medical treatment	47.5 months

### The AIP and CAD severity in populations without established CAD

In populations without established CAD, four studies evaluated the relationship between AIP levels and coronary artery plaque progression [25–28]. Additionally, two studies evaluated the relationship between AIP levels and the presence of multivessel lesions [17, 18], while one study investigated the association between AIP levels and coronary artery calcification [15]. Details of these studies are available in Table 1.

**Fig. 2:**
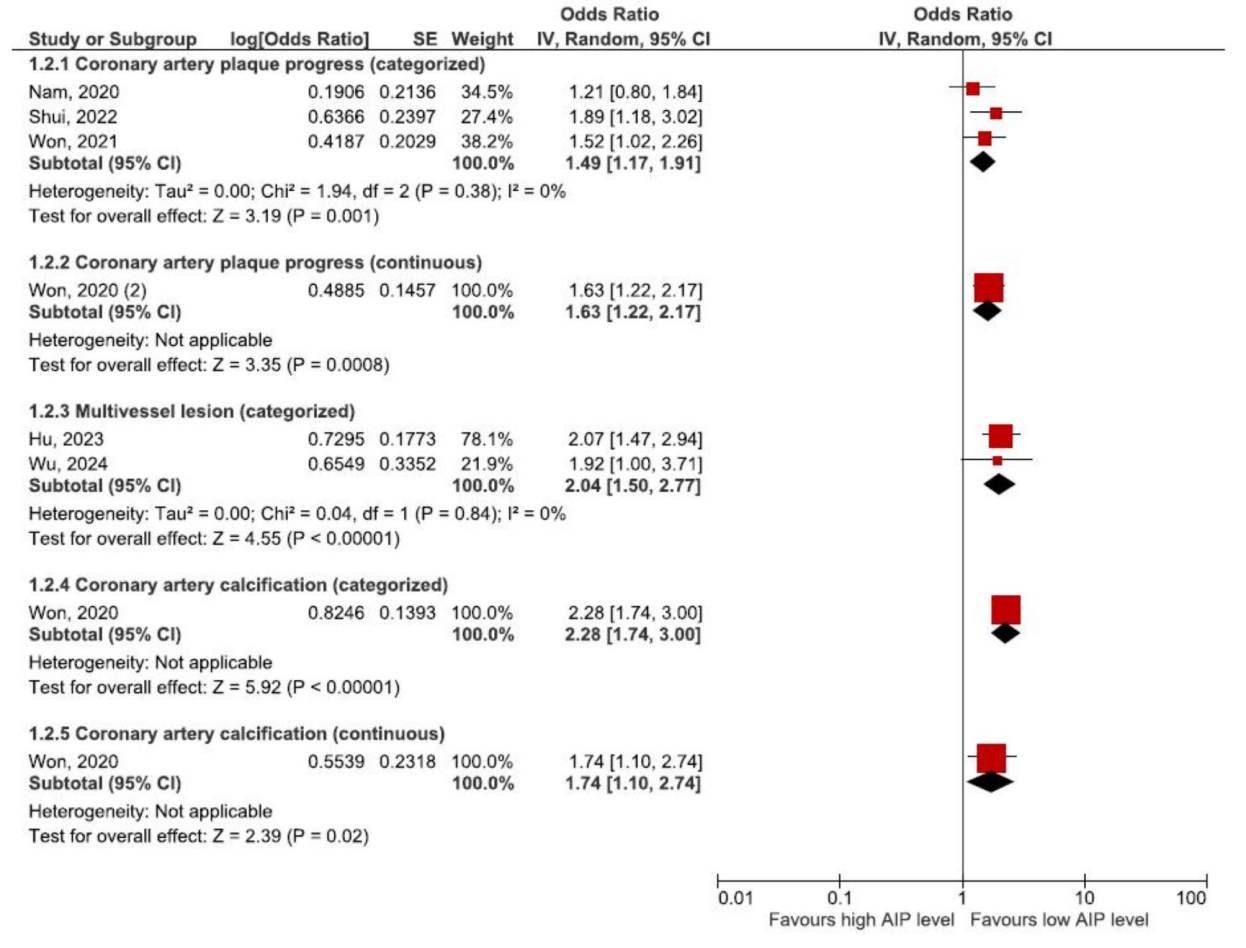
Forest plots showing the pooled result of meta-analysis of CAD severity in populations without established CAD

One study [15] revealed that in asymptomatic patients, higher AIP levels were associated with an increased likelihood of coronary artery calcification (OR: 2.28, 95% CI 1.74–3.00, *P* < 0.00001).The pooled results of two studies [17, 18] demonstrated that individuals with higher AIP levels were significantly more likely to have multivessel CAD compared to those with lower AIP levels (OR: 2.04, 95% CI 1.50–2.77, I^2^ = 0%, *P* < 0.00001). The meta-analysis of three studies [25–27] showed that individuals with higher AIP had a significantly increased risk of plaque progression (OR: 1.49, 95% CI 1.17–1.91, I^2^ = 0%, *P* = 0.001). This association remained consistent when AIP was analyzed as a continuous variable in another study (OR per 1-unit increment of AIP: 1.63, 95% CI 1.22–2.17, *P* = 0.02). Figure 2 provides the details of the analyses.

### The AIP and cardiovascular events in populations without established CAD

In this meta-analysis, seven studies evaluated the risks of MACE [9, 29–34], six studies assessed CVD-specific mortality [9, 29, 32, 35–37], six studies examined all-cause mortality [29, 35–39], six studies investigated the risks of stroke [29, 32, 40–43], and four studies evaluated the risks of MI [29, 32, 44, 45] in populations without established CAD. The details of these studies are provided in Table 1.

**Fig. 3:**
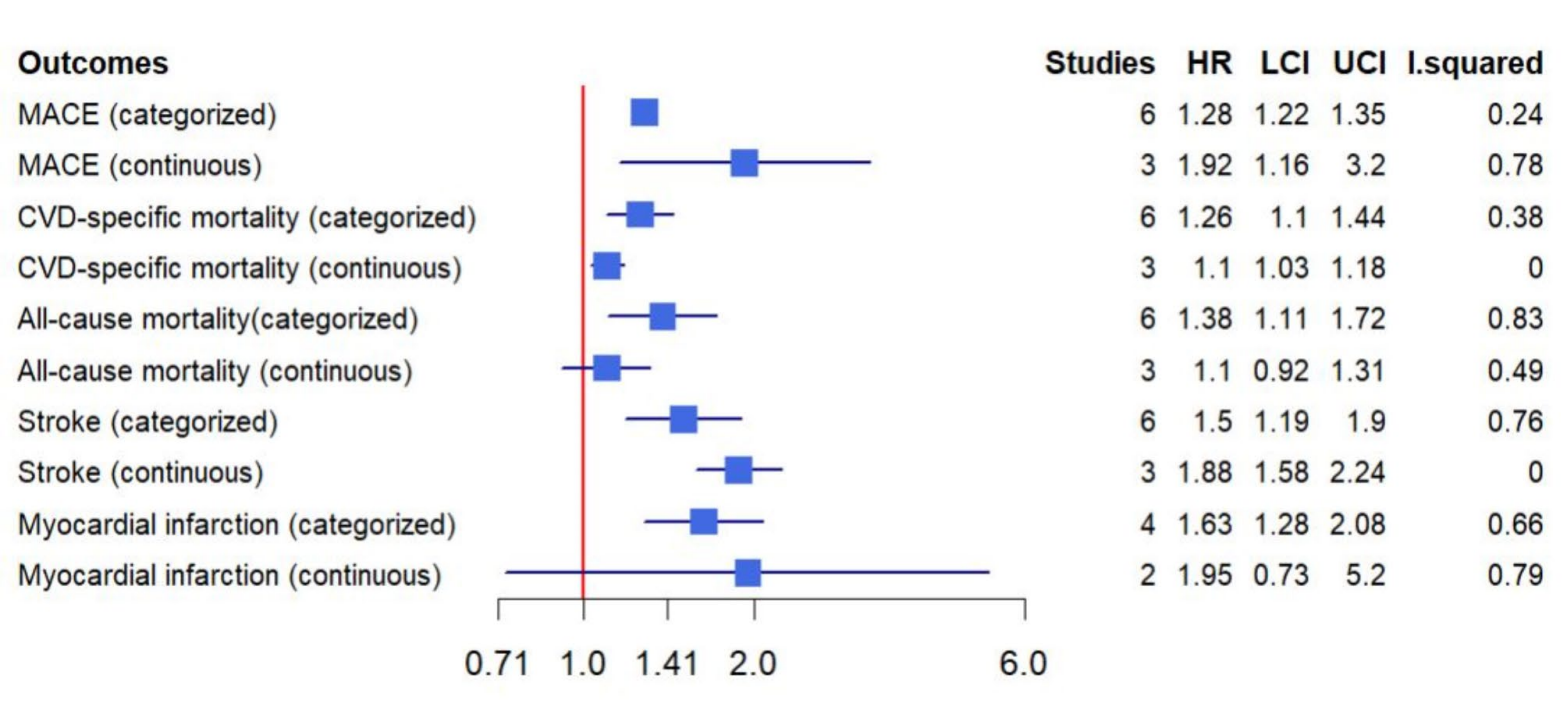
The pooled result of the incidence rate of the adverse outcomes in populations without established CAD

The meta-analysis of six studies revealed that individuals with higher AIP levels had a significantly increased risk of MACE compared to those with lower AIP levels (HR: 1.28, 95% CI 1.22–1.35, I^2^ = 24%, *P* < 0.00001). This association was consistent when AIP was analyzed as a continuous variable in three studies (HR per 1-unit increment of AIP: 1.92, 95% CI 1.16–3.20, I^2^ = 78%, *P* = 0.01). Figure 3 and Supplementary eFigure 2 show the results for other outcomes.

### The AIP and CAD severity in populations with established CAD

In patients with CAD undergoing CAG, higher AIP levels were associated with an increased risk of coronary total occlusion [46] (OR: 5.23, 95% CI 4.36–6.26, *P* < 0.00001) and poor coronary collateral circulation [47] (OR: 4.47, 95% CI 2.77–7.22, *P* < 0.00001). In patients with acute coronary syndrome (ACS) undergoing percutaneous coronary intervention (PCI), higher AIP levels significantly increased the risk of in-stent restenosis [48] (OR: 5.23, 95% CI 4.36–6.26, *P* < 0.00001) and stent-thrombosis [49] (OR: 4.47, 95% CI 2.77–7.22, *P* < 0.00001). In patients with ST-elevation MI undergoing PCI, higher AIP levels were associated with the no-reflow phenomenon [50] (OR: 16.79, 95% CI 1.02–277.03, *P* = 0.04). The details of the studies are presented in Table 1 and Supplementary eFigure 3.

### The AIP and cardiovascular events in populations with established CAD

**Fig. 4:**
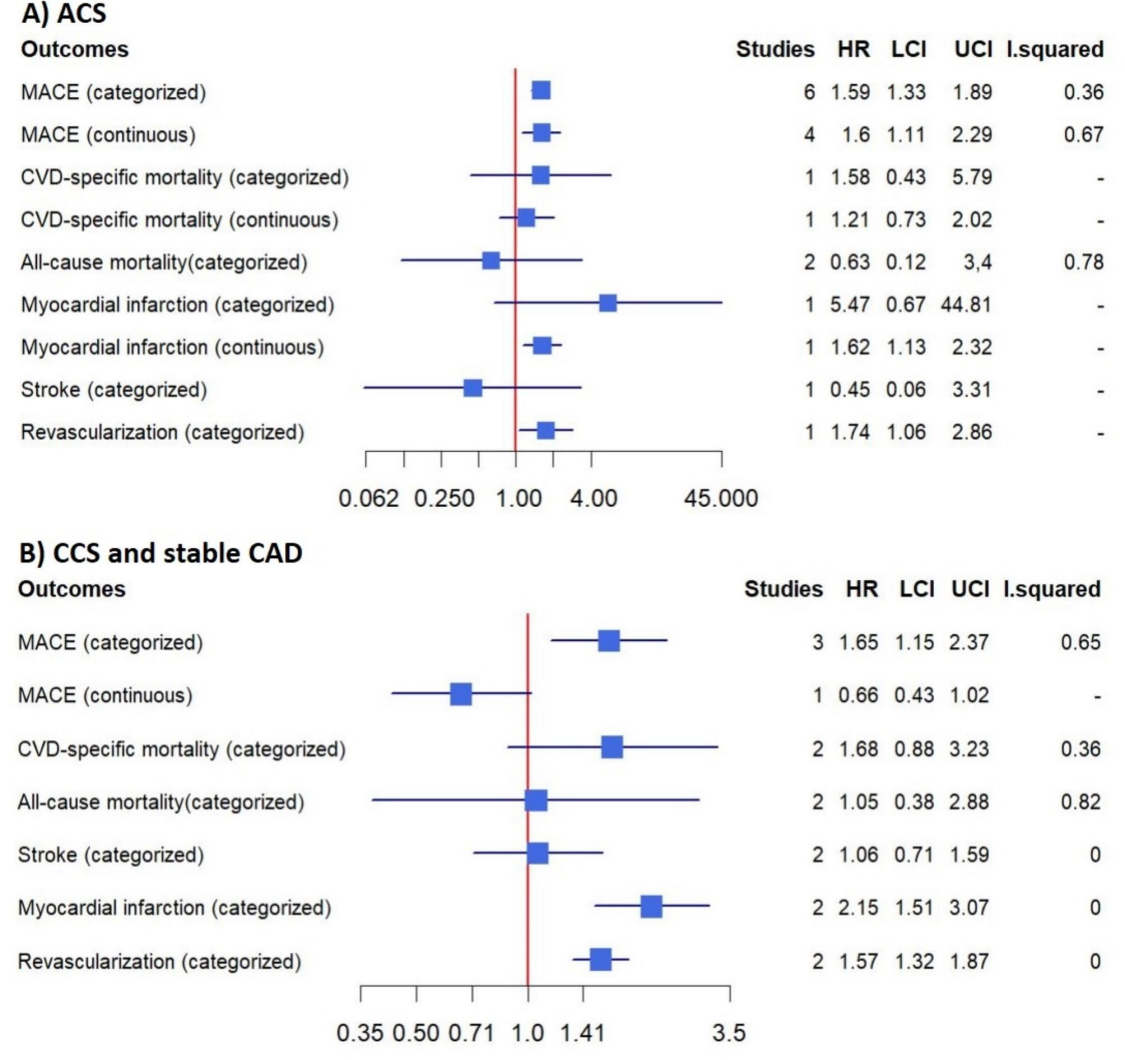
The pooled result of the incidence rate of the adverse outcomes in populations with established CAD

In this meta-analysis, a total of nine studies evaluated the prognosis of patients with ACS [5, 7, 51–57], and four studies assessed the prognosis of patients with chronic coronary syndrome (CCS) or stable CAD [5, 8, 58, 59]. The details of the studies are presented in Table 1. In patients with ACS, higher AIP levels were associated with an increased risk of MACE compared with lower AIP levels [7, 51–55] (HR: 1.59, 95% CI 1.33–1.89, I^2^ = 86%, *P* < 0.00001). This association was also observed when AIP was analyzed as a continuous variable [5, 7, 51, 56] (HR per 1-unit increment of AIP: 1.60, 95% CI 1.11–2.29, I^2^ = 67%, *P* = 0.01). In patients with CCS, higher AIP levels significantly increased the risk of MACE [8, 58, 59] (HR: 1.65, 95% CI 1.15–2.37, I^2^ = 65%, *P* = 0.007), but this association was non-significant when AIP was analyzed as a continuous variable [5] (HR per 1-unit increment of AIP: 0.66, 95% CI 0.43–1.02, *P* = 0.06). Figure 4 and Supplementary eFigure 5 show the results for other outcomes.

### Sensitivity analysis, subgroup analysis and publication bias

For categorized outcomes that showed statistically significant results in the meta-analyses, sensitivity analysis, in which one study was omitted at a time, consistently demonstrated significant results. However, this pattern was not observed for MACE in patients with CCS or stable CAD, and for coronary artery plaque progression. On the other hand, for statistically significant continuous outcomes, less consistency was observed across the sensitivity analyses. The results remained consistent only for stroke in populations without established CAD and the risk of CAD. Supplementary eTable 5 shows the results of these analyses.

The subgroup analyses generally showed no significant differences based on gender or diabetes status for the majority of outcomes (P-value for subgroup differences > 0.05). However, subgroup analyses indicated that the relationship between AIP and MACE was stronger among diabetic patients with ACS. A similar stronger association between AIP and all-cause mortality was observed among diabetic patients with stable CAD or CCS. The details of these analyses can be found in Supplementary eTable 4.

Supplementary eFigure 6 shows the funnel plot for the association between AIP levels and CAD risk. Upon visual examination, the plot appears symmetrical, indicating low publication bias. This finding was confirmed by Egger’s regression test (*p* = 0.69). Egger’s regression tests could not be performed for other outcomes due to fewer than ten studies being available for each outcome.

## Discussion

AIP is a novel logarithmic index that combines fasting triglyceride levels and HDL-C levels. The results of this meta-analysis reveal that individuals with higher AIP levels are more likely to have CAD, more severe coronary lesions, and a worse prognosis compared to those with lower AIP levels after adjusting for conventional cardiovascular risk factors. These findings remained consistent when AIP was analyzed as a continuous variable for most of the outcomes.

### Higher AIP levels are associated with higher CAD risk

For the first time, Dobiášová et al. [2] proposed AIP as a marker of plasma atherogenicity, noting that it inversely correlates with low-density lipoprotein cholesterol (LDL-C) particle size. Specifically, small dense low-density lipoprotein (sdLDL), a subtype of LDL-C, has a smaller particle size and is more prone to invading and depositing on arterial walls compared to regular LDL [60]. Moreover, sdLDL particles are more easily oxidized, and oxidized LDL-C is phagocytized by macrophages, which differentiate into foam cells, contributing to atherogenesis. The mechanisms proposed to justify the greater atherogenic potential of sdLDL particles include lipid peroxidation, the expression of adhesion molecules on endothelial cells, and activation of oxygen radicals. All these processes work together to further promote CAD pathogenesis [61, 62]. In support of this, numerous studies have demonstrated a strong association between higher sdLDL levels and the development of CAD [63]. However, because of the high costs and complex techniques involved in its detection, measuring sdLDL is rarely used in clinical practice. It is noteworthy that AIP has been shown to serve as a surrogate for sdLDL. An increase in AIP is a good indicator of reduced particle size of LDL-C and thus increased proportion of sdLDL particles [2]. Apart from sdLDL, remnant lipoprotein cholesterol (RLP-C) is another important component associated with higher AIP [26]. Genetic studies utilizing Mendelian randomization and single-nucleotide polymorphisms have clearly established that RLP-C is an independent causal factor for ischemic heart disease [64]. All these findings further emphasize the importance of AIP as a marker of atherogenic risk. Our study provides additional evidence to sustain the association between AIP and CAD, thereby reinforcing the notion of AIP as a reliable marker for atherosclerotic risk.

### Higher AIP levels are associated with more severe coronary lesions

In addition to sdLDL and RLP-C, elevated AIP levels are also closely associated with higher uric acid levels [65]. All three of these parameters [66–68] contribute to the development of CAD and the exacerbation of atherosclerosis. The severity of CAD is closely related to several factors, including the extent of coronary artery calcification, plaque progression, and the presence of multivessel coronary lesions. Furthermore, it correlates with occurrences such as the no-reflow phenomenon, coronary total occlusion, the presence of poor coronary collateral circulation, and the development of in-stent restenosis and stent thrombosis following the placement of stent. Our study suggests that higher AIP levels are associated with more severe CAD in individuals both with and without an existing CAD diagnosis. In support of this, the study done by Won et al. [15], found that AIP level higher than 0.28 is a significant predictor of the presence of coronary artery calcium score (CACS) above 100 in the general population, with a sensitivity of 70.5% and a specificity of 42.6%. Additionally, several studies [26–28] have concluded that higher AIP levels are associated with coronary artery plaque progression. However, it is worthy of note that this association was not observed in individuals with baseline CACS above 100 [28], that this association may differ based on the severity of existing calcification. Moreover, multivessel coronary lesions negatively impact the prognosis of CAD and increase the complexity of PCI. Two studies [17, 18] reported that the presence of multivessel coronary lesions is correlated with elevated AIP levels. However, it should be emphasized that, in the study conducted by Won et al. [18], no correlations were present in either females or elderly subpopulations, indicating a divergence in the relationship across demographics. In settings of PCI, in-stent restenosis and stent thrombosis pose significant challenges, and higher AIP levels are positively correlated with these adverse events following stent placement [48, 49]. In addition, elevated AIP is associated with the no-reflow phenomenon after PCI [50, 69, 70]. It is also noteworthy that there is a dose-response relationship between AIP and poor coronary collateral circulation [47], thereby further illustrating that AIP plays multiple roles in CAD progression and severity.

### Higher AIP levels are associated with poor prognosis

Elevated AIP levels predict a worse cardiovascular prognosis in individuals both with and without established CAD. This relationship can be explained in two ways. Firstly, our study and several others have shown that higher AIP levels are associated with structural and functional abnormalities of the coronary artery, leading to poor outcomes. The no-reflow phenomenon, for instance, is associated with reduced myocardial salvage and is a strong predictor of 5-year mortality following PCI [71]. In contrast, well-developed coronary collateral circulation reduces myocardial infarct size and decreases ventricular aneurysm formation, whereas poor coronary collateral circulation undermines this protective effect [72]. Secondly, elevated AIP is often accompanied by multiple risk factors for CVD. A cross-sectional study in Mexico found that higher AIP values are associated with hyperlipidemia, hypertension, and metabolic syndrome [73]. Similarly, Yin et al. [4] identified an inverse L-shaped relationship between AIP and insulin resistance, which has a known causal connection to CVD. Insulin resistance contributes to CVD generation via two mechanisms [74]: (1) Atherosclerotic plaque formation and (2) ventricular hypertrophy and diastolic dysfunction.

This meta-analysis further highlights that in populations without established CAD, elevated AIP increases the risk of MACE, CVD-specific mortality, and stroke in both categorized and continuous AIP analyses. In specific conditions, such as patients undergoing peritoneal dialysis, elevated AIP predicts an increased risk of mortality [36]. For those with antibody-associated vasculitis [40] and obstructive sleep apnea [44], elevated AIP is linked to a higher incidence of stroke and MI, respectively. In contrast, given the limited number of studies conducted in populations with established CAD, the findings were less consistent. Only patients with ACS exhibited a significantly elevated risk of MACE in both continuous and categorized analyses. For other endpoints, the results were either not statistically significant, showed inconsistencies between analyses, or lacked a second study with an opposing analysis for consistency verification. This discrepancy is probably due to the lower number of studies and participants, which reduces statistical power necessary to detect significant differences.

An interesting finding from a retrospective cohort study by Wang et al. [6], indicated that AIP was a significant predictor of adverse events in patients with ACS who underwent PCI with LDL-C levels below 1.8mmol/L. This suggests that AIP holds additional prognostic information that is beyond what the traditional CVD risk factors provide. This was further supported by two other studies [52, 54] showing that AIP is positively associated with adverse events in ACS patients undergoing PCI, even after adjusting for SYNTAX and GRACE risk scores. These findings imply that the incorporation of AIP into existing scoring systems would probably improve the predictive accuracy for cases of CAD management.

### Current stage and clinical implications

To our knowledge, our study is the first systematic review and meta-analysis to thoroughly summarize the studies on AIP and CAD, offering an evidence-based foundation for clinical practice. While AIP is not explicitly involved in clinical guidelines, the role of managing triglyceride and HDL-C levels in the prevention of CVD is reflected in some guidelines. Recently, the American Diabetes Association (ADA) published its 2024 “Standards of Care in Diabetes” [75], suggesting that patients with elevated triglyceride levels (≥ 150 mg/dL [≥ 1.7 mmol/L]) and/or low HDL-C (< 40 mg/dL [< 1.0 mmol/L] for men and < 50 mg/dL [< 1.3 mmol/L] for women) should enhance lifestyle interventions and optimize glycemic control. Since AIP is calculated using these parameters, managing triglyceride and HDL-C naturally reduces AIP levels. This meta-analysis demonstrates that elevated AIP is associated with a higher risk, greater severity, and poorer prognosis of CAD in both individuals with and without established CAD. Thus, controlling factors associated with AIP, including triglycerides and HDL-C, can help substantially reduce the occurrence of CVD and improve prognosis. The simplicity of AIP measurement makes it particularly valuable for low-resource settings, where comprehensive laboratory tests may not be available. From a public health perspective, risk stratification based on AIP would lead to more efficient treatment strategies that could help mitigate the burden of cardiovascular diseases in societies.

AIP has emerged as a promising new biomarker for evaluating CAD risk, severity, and prognosis. Based on the results of this systematic review, some knowledge gaps still exist that further research in these areas could lead to a better understanding of the role of AIP in CAD. Firstly, 42 out of 52 studies included in this review were conducted on populations with Asian ethnicity. Therefore, additional studies are needed to clarify the predictive value of AIP in other ethnicities. Secondly, we identified only two studies [52, 54] that evaluated the predictive value of AIP after adjusting for commonly used risk scores. Further research evaluating the incorporation of AIP into existing scoring systems could provide valuable insights into its future applications as a biomarker. Thirdly, due to the lack of access to patient-level data, there were only a few studies included in the subgroup analyses, which reduced the statistical power needed to detect meaningful differences. Despite these limitations, subgroup analyses revealed a stronger association between AIP and some outcomes in diabetic patients. Further studies are needed to clarify and provide mechanistic explanations for this discrepancy. Finally, the results in patients with stable CAD or CCS were less robust and sensitivity analyses demonstrated inconsistencies. We need more studies to evaluate the predictive value of AIP in patients with CCS or stable CAD in order to draw definitive conclusions.

### Limitations

The present study has several limitations. First, as a meta-analysis of observational studies, causation cannot be proven, limiting the strength of evidence. Second, this meta-analysis generated evidence from study-level data only, which precludes the possibility of more subgroup analyses that would be possible with patient-level data. Third, despite controlling for confounders, different studies adjusted for different sets of confounders, making the presence of residual confounding inevitable and leading to some degree of bias. Fourth, outcomes were inconsistent across the 51 studies we included, leading to a small number of studies for each outcome. This may reduce the statistical power necessary to detect significant differences. Fifth, differences in the definition of the endpoints, such as MACE, may affect the interpretation of the results. Finally, most of the data were obtained from individuals of Asian ethnicity, and further studies are needed to evaluate the predictive performance of AIP in other races.

## Conclusion

AIP is a straightforward yet effective mathematical formula that has proven to be a valuable indicator for determining the risk of CAD, evaluating its severity, and predicting prognosis. Individuals with higher AIP are more likely to develop CAD, have more severe CAD, and show a poorer prognosis compared to individuals with lower AIP. These findings have significant implications for risk stratification and management of both individuals with CAD and those without previously known CAD. Further research is essential to validate and expand upon these findings.

## Electronic supplementary material

Below is the link to the electronic supplementary material.

Supplementary Material 1

## Data Availability

No datasets were generated or analysed during the current study.

## Abbreviations

CVD: Cardiovascular disease
CAD: Coronary artery disease
MI: Myocardial infarction
AIP: Atherogenic index of plasma
TG: Triglyceride
HDL-C: High-density lipoprotein cholesterol
PRISMA: Preferred Reporting Items for Systematic Reviews and Meta-Analyses
OR: Odds ratio
HR: Hazard ratio
CI: Confidence interval
CAG: Coronary angiography
MACE: Major adverse cardiovascular events
NOS: Newcastle–Ottawa Scale
ACS: Acute coronary syndrome
PCI: Percutaneous coronary intervention
CCS: Chronic coronary syndrome
LDL-C: Low-density lipoprotein cholesterol
SdLDL: Small dense low-density lipoprotein
RLP-C: Remnant lipoprotein cholesterol
CACS: Coronary artery calcium score
ADA: American Diabetes Association
