# Supplementary material for: Atherogenic index of plasma and coronary artery disease: a systematic review and meta-analysis of observational studies
Source: Cardiovasc Diabetol. 2025 Jan 22;24:35. doi: 10.1186/s12933-025-02582-2 (PMC11756160; doi:10.1186/s12933-025-02582-2)

**Supplementary material**

**eTable 1. Search strategy**

**eTable 2. Definition of the endpoints**

**eTable 3. NOS of the included studies**

**eFigure 1. PRISMA 2020 flow diagram**

**eFigure 2. Adverse events in populations without established CAD**

**eFigure 3. CAD severity in populations with established CAD**

**eFigure 4. Adverse events in patients with ACS**

**eFigure 5. Adverse events in patients with CCS or stable CAD**

**eFigure 6. Funnel plot for risk of CAD**

**eTable 4. Subgroup analyses**

**eTable 5. Sensitivity analyses**

**Confirmation of Publication and Licensing Rights – Open Access**

**eTable 1. Search strategy**

| **Data base** | **Method** | **Result** |
| --- | --- | --- |
| **Pubmed** |  |  |
| #1 | ‘atherogenic index’[Title/Abstract] OR ‘atherogenic index of plasma’[Title/Abstract] | 2,235 |
| #2 | ‘cardiovascular disease’ OR ‘cardiovascular diseases’ OR ‘CVD’ OR ‘coronary heart disease’ OR ‘CHD’ OR ‘coronary artery disease’ OR ‘CAD’ OR ‘myocardial infarction’ OR ‘MI’ OR ‘coronary arteriosclerosis’ OR ‘heart attack’ OR ‘heart failure’ OR ‘HF’ OR ‘heart decompensation’ OR ‘atrial fibrillation’ OR ‘AF’ OR ‘sudden cardiac death’ OR ‘SCD’ OR ‘arrhythmia’ OR ‘cardiomyopathy’ OR ‘hypertrophic cardiomyopathy’ OR ‘HCM’ OR ‘dilated cardiomyopathy’ OR ‘DCM’ OR ‘mortality’ OR ‘death’ OR ‘cardiovascular death’ OR ‘all-cause mortality’ | 7,051,911 |
| #3 | #1 AND #2 | 1,335 |
| **Embase** |  |  |
| #1 | 'atherogenic index':ti,ab OR 'atherogenic index of plasma':ti,ab | 3,234 |
| #2 | ‘cardiovascular disease’ OR ‘cardiovascular diseases’ OR ‘CVD’ OR ‘coronary heart disease’ OR ‘CHD’ OR ‘coronary artery disease’ OR ‘CAD’ OR ‘myocardial infarction’ OR ‘MI’ OR ‘coronary arteriosclerosis’ OR ‘heart attack’ OR ‘heart failure’ OR ‘HF’ OR ‘heart decompensation’ OR ‘atrial fibrillation’ OR ‘AF’ OR ‘sudden cardiac death’ OR ‘SCD’ OR ‘arrhythmia’ OR ‘cardiomyopathy’ OR ‘hypertrophic cardiomyopathy’ OR ‘HCM’ OR ‘dilated cardiomyopathy’ OR ‘DCM’ OR ‘mortality’ OR ‘death’ OR ‘cardiovascular death’ OR ‘all-cause mortality’ | 5,457,774 |
| #3 | #1 AND #2 | 1,457 |
| **Web of science** |  |  |
| #1 | (TI="atherogenic index" OR AB="atherogenic index") OR (TI="atherogenic index of plasma" OR AB="atherogenic index of plasma") | 2,529 |
| #2 | ALL=("cardiovascular disease" OR "cardiovascular diseases" OR CVD OR "coronary heart disease" OR CHD OR "coronary artery disease" OR CAD OR "myocardial infarction" OR MI OR "coronary arteriosclerosis" OR "heart attack" OR "heart failure" OR HF OR "heart decompensation" OR "atrial fibrillation" OR AF OR "sudden cardiac death" OR SCD OR arrhythmia OR cardiomyopathy OR "hypertrophic cardiomyopathy" OR HCM OR "dilated cardiomyopathy" OR DCM OR mortality OR death OR "cardiovascular death" OR "all-cause mortality") | 5,724,370 |
| #3 | #1 AND #2 | 1,164 |

**eTable 2. Definition of the endpoints**

| **Study** | **Definition** |
| --- | --- |
| Dong, 2024 | Coronary calcium score was graded according to the Rentrop scoring system |
| Liu, 2024 | MACE: stroke, MI and cardiovascular mortality |
| Qin, 2024 | heart disease and cerebrovascular disease mortality |
| Qu, 2024 | Self-reported stroke was assessed with the following questions: “Have you been diagnosed with stroke by a doctor”; “Have you been diagnosed with stroke by a doctor since the last follow-up visit?”; “Compared to when we interviewed you last time, is your stroke condition better, about the same as it was then, or worse?” |
| Shui, 2022 | angiographic progression was defined as the Gensini score (GS) rate of change per year >1 point |
| Wu, 2024 | CAD is characterized by a luminal constriction of ≥ 50% in one principal coronary artery. The severity of CAD is contingent upon the quantity of narrowed coronary arteries. Narrowing of ≥ 50% in the left main artery is also considered multi-vessel CAD. |
| Yu, 2024 | The start of the follow-up for all ADHF patients is set at the time of admission, and their 30-day survival status is obtained by trained medical workers through text messages, phone calls, and face-to-face follow-ups in outpatient and inpatient settings. |
| Zhang, 2024 | The diagnosis of MI was determined by the patient’s clinical symptoms, electrocardiogram, and dynamic changes of myocardial enzyme following the World Health Organization’s Multinational Monitoring of Trends and Determinants in Cardiovascular Disease criteria |
| Zhang, 2024 (2) | Stroke subtypes, including IS (163) and ICH (161–162), were defined using the International Classification of Diseases-10th Revision (ICD-10) codes. Stroke diagnosis was routinely conducted using computed tomography and/or magnetic resonance imaging, and the diagnostic criteria were consistent across all participating hospitals |
| Zhi, 2024 | nonfatal myocardial infarction, nonfatal stroke, and/or death from cardiovascular causes |
| Abdu, 2024 | Non-obstructive CAD in MINOCA was categorized as the presence of vessels with stenosis ranging from 0 % to 50 %. Primary clinical endpoint included MACE, which encompassed cardiac deaths, nonfatal MI, heart failure, stroke, and angina rehospitalization. |
| Han, 2024 | TIMI 0 forward blood flow of the occluded vessel segment, and with the occlusion lasting for over 3 months |
| Won, 2024 | MACE: all-cause death, myocardial infarction (MI), or cerebrovascular accident (CVA) |
| Deng, 2023 | Cardiovascular mortality was defined according to the International Classification of Diseases Clinical Modification |
| Hu, 2023 | CAD was considered to be present if there was 50% or more stenosis in one of the major coronary arteries. A multivessel lesion was defined when two or more major coronary arteries had 50% or more stenosis. |
| Tamosiunas, 2023 | CVD-specific mortality was categorized using codes I00-I99 |
| Won, 2020 (2) | CAC progression was defined using the SQRT method; specifically, as a difference ≥ 2.5 between the square roots (√) of the baseline and follow-up CACSs (Δ√transformed CACS), with consideration of the inter-scan variability |
| Alifu, 2023 | MACE: Cardio-vascular death, Ischemia-driven revascularization, nonfatal MI, heart failure, and nonfatal stroke. |
| Kan, 2023 | MACCE: all-cause mortality, non-fatal ischemic stroke, non-fatal spontaneous myocardial infarction, and unplanned repeat revascularization |
| Liu, 2023 | MACE: cardiac death, refractory angina, and non-fatal myocardial infarction (MI) |
| Ozen, 2023 | MACE: cardiac death, nonfatal myocardial infarction, target vessel revascularization (TVR), congestive heart failure (CHF), and nonfatal stroke. |
| Wang, 2023 | MACCE: cardiac death, non-fatal myocardial infarction (MI), non-fatal stroke, and unplanned repeat revascularization. All deaths were considered of cardiac origin unless a noncardiac origin was established clinically or at autopsy. Non-fatal MI was defined in accordance with the fourth universal definition of MI. Non-fatal stroke is defined as any acute new neurological deficit lasting longer than 24 h accompanied by neuroimaging evidence of brain ischemia or bleeding. Unplanned repeat revascularization was defined as any revascularization driven by angina or ischemia, either PCI or coronary artery bypass graft, of any segment of the target or nontarget vessel. |
| Cai, 2022 | Death certificates and hospital records confirmed fatal MI. For out-of-hospital deaths, follow-up data were obtained by contacting family members by telephone |
| Hang, 2022 | MACE: myocardial infarction (MI), non-MI acute coronary syndrome, stroke, cardiovascular mortality, and heart failure. |
| Kim, 2022 | MACE: nonfatal myocardial infarction (MI), non-fatal stroke, and CV mortality |
| Mahdaviroshan, 2022 | angiographically confirmed CAD in accordance with “ESC 2019 guidelines for the diagnosis and management of chronic coronary syndromes |
| Mangalesh, 2022 | MACE: death, hospitalization for heart failure, myocardial infarction and revascularization including percutaneous coronary intervention. |
| Zhu, 2022 | The in-stent restenosis was defined as a lumen diameter stenosis ≥ 50% occurred at the segment inside the stent or involving its 5 mm-edges. |
| Abacıoğlu, 2022 |  |
| Qiao, 2022 | MACCE: all-cause mortality, non-fatal MI, non-fatal ischemic stroke, or unplanned repeat revascularization |
| Zheng, 2022 | MACCE: cardiac death, target vessel revascularization (TVR), and non-fatal myocardial infarction (MI). The definition for non-fatal myocardial infarction in this study was defined as Type I myocardial infarction after PCI. Cardiac death was defined as any death which did not have a clear extracardiac origin, or myocardial infarction in accordance with guidelines. Target vessel revascularization was defined as angina or ischemia referable to the target vessel requiring repeat PCI or Coronay Artery Bypass Graft, otherwise known as CABG |
| Fu, 2021 | MACE: nonfatal myocardial infarction (MI), nonfatal stroke, and/or death from cardiovascular causes |
| Hongbing, 2021 |  |
| Liu, 2021 | CTO was defined as follows: (1) an occlusion lasting for more than 3 months based on the first onset of angina pectoris, previous angiogram findings, and previous infarction, and (2) TIMI Grade 0. |
| Sadeghi, 2021 | MACE: MI, ACS, stroke or heart failure |
| Won, 2021 | Rapid plaque progression (RPP) was defined as the change of percentage atheroma volume (PAV) ≥1.0%/year |
| Si, 2021 | coronary artery calcium score ≥ 100 |
| Wang, 2021 | Coronary Artery Disease (CAD) is diagnosed based on coronary angiography results using the SYNTAX Score (SS), with coronary artery stenosis ≥50% in vessels ≥1.5 mm diameter, combined with WHO criteria. Severity is classified as low (SS ≤22), intermediate (SS 23-32), or high (SS ≥33), with SS ≥23 considered high risk. |
| Zhou, 2021 | The definition of CAD was ≥50% diameter stenosis of at least 1 major coronary artery according to angiograms |
| Refaat, 2021 | TIMI<3 |
| Ahn, 2020 | cerebral infarction, transient ischemic attack, and cerebral hemorrhage |
| Nam, 2020 | either incident CAC in a CAC-free population at baseline, or an increase of ≥2.5 units between the square roots of the baseline and follow-up coronary artery calcium scores (CACS) in subjects with detectable CAC at baseline. |
| Wang, 2020 |  |
| Won, 2020 | obstructive coronary plaque (OCP) defined as coronary plaque with ≥50% luminal narrowing |
| Ma, 2020 | MACCE: death from any cause, non-fatal spontaneous myocardial infarction (MI), non-fatal ischemic stroke, and unplanned repeat revascularization |
| Qin, 2020 | MACCE: a combination of cardiogenic death, myocardial infarction, repeated revascularization, and stroke |
| Wu, 2018 | CAD was defined in accordance with the 1979 WHO diagnostic criteria, and all of the patients underwent a CAG examination. |
| Cai, 2017 | CAD was defined in accordance with the 1979 WHO diagnostic criteria. |
| Ni, 2017 | Diagnostic criteria of CHD set by the World Health Organization, i.e. an individual with CHD should have at least one coronary stenosis ≥50% |
| Anggoro, 2016 | Death from all etiologies during intensive hospitalization |
| Onat, 2010 | Diagnosis of nonfatal CHD was determined on the basis of the presence of angina pectoris, of a history of myocardial infarction with or without accompanying Minnesota codes of the electrocardiogram, or by history of myocardial revascularization. CHD death comprised death resulting from heart failure of coronary origin and a fatal coronary event. |

**eTable 3. NOS of the included studies**

| **Study** | **Selection** | | | | **Comparability** | **Outcome** | | | **Overall** |
| --- | --- | --- | --- | --- | --- | --- | --- | --- | --- |
|  | **1** | **2** | **3** | **4** | **1** | **1** | **2** | **3** |  |
| Dong, 2024 | 1 | 1 | 1 | 1 | 2 | 1 | 0 | 0 | **7** |
| Liu, 2024 | 1 | 1 | 1 | 1 | 2 | 1 | 1 | 0 | **8** |
| Qin, 2024 | 1 | 1 | 1 | 0 | 2 | 1 | 1 | 1 | **8** |
| Qu, 2024 | 1 | 1 | 1 | 1 | 1 | 1 | 1 | 1 | **8** |
| Shui, 2022 | 0 | 1 | 1 | 1 | 1 | 1 | 0 | 1 | **6** |
| Wu, 2024 | 1 | 0 | 0 | 1 | 2 | 1 | 1 | 0 | **6** |
| Yu, 2024 | 1 | 1 | 1 | 1 | 2 | 1 | 0 | 1 | **8** |
| Zhang, 2024 | 1 | 1 | 1 | 1 | 2 | 1 | 1 | 0 | **8** |
| Zhang, 2024 (2) | 1 | 1 | 1 | 1 | 2 | 1 | 1 | 1 | **9** |
| Zhi, 2024 | 1 | 1 | 1 | 1 | 2 | 1 | 1 | 1 | **9** |
| Abdu, 2024 | 1 | 1 | 1 | 1 | 1 | 1 | 0 | 1 | **7** |
| Han, 2024 | 0 | 1 | 1 | 1 | 2 | 1 | 1 | 1 | **8** |
| Won, 2024 | 1 | 1 | 1 | 1 | 2 | 1 | 1 | 1 | **9** |
| Deng, 2023 | 1 | 1 | 1 | 1 | 2 | 1 | 0 | 0 | **7** |
| Hu, 2023 | 1 | 0 | 0 | 1 | 2 | 1 | 1 | 0 | **6** |
| Tamosiunas, 2023 | 1 | 1 | 0 | 0 | 2 | 1 | 1 | 1 | **7** |
| Won, 2020 (2) | 0 | 0 | 1 | 0 | 2 | 1 | 1 | 1 | **6** |
| Alifu, 2023 | 0 | 1 | 1 | 1 | 1 | 1 | 0 | 1 | **6** |
| Kan, 2023 | 0 | 1 | 1 | 1 | 2 | 1 | 0 | 1 | **7** |
| Liu, 2023 | 1 | 1 | 0 | 1 | 2 | 1 | 0 | 0 | **6** |
| Ozen, 2023 | 1 | 1 | 1 | 1 | 2 | 1 | 0 | 0 | **7** |
| Wang, 2023 | 1 | 1 | 1 | 1 | 2 | 1 | 0 | 1 | **8** |
| Cai, 2022 | 1 | 1 | 1 | 1 | 2 | 1 | 1 | 1 | **9** |
| Hang, 2022 | 1 | 1 | 1 | 1 | 2 | 1 | 0 | 0 | **7** |
| Kim, 2022 | 1 | 1 | 1 | 1 | 2 | 1 | 0 | 0 | **7** |
| Mahdaviroshan, 2022 | 1 | 0 | 0 | 0 | 2 | 1 | 0 | 0 | **4** |
| Mangalesh, 2022 | 1 | 1 | 0 | 1 | 2 | 1 | 0 | 0 | **6** |
| Zhu, 2022 | 1 | 1 | 1 | 1 | 1 | 1 | 1 | 1 | **8** |
| Abacıoğlu, 2022 | 0 | 1 | 0 | 1 | 1 | 1 | 0 | 0 | **4** |
| Qiao, 2022 | 1 | 1 | 1 | 1 | 1 | 1 | 0 | 1 | **7** |
| Zheng, 2022 | 0 | 1 | 1 | 1 | 2 | 1 | 0 | 1 | **7** |
| Fu, 2021 | 1 | 1 | 1 | 1 | 2 | 1 | 1 | 0 | **8** |
| Hongbing, 2021 | 0 | 1 | 0 | 1 | 2 | 0 | 1 | 1 | **6** |
| Liu, 2021 | 1 | 1 | 1 | 1 | 2 | 1 | 1 | 0 | **8** |
| Sadeghi, 2021 | 1 | 1 | 1 | 0 | 2 | 1 | 1 | 0 | **7** |
| Won, 2021 | 0 | 1 | 1 | 1 | 2 | 1 | 1 | 1 | **8** |
| Si, 2021 | 1 | 0 | 0 | 1 | 2 | 1 | 1 | 0 | **6** |
| Wang, 2021 | 1 | 1 | 1 | 0 | 1 | 1 | 1 | 1 | **7** |
| Zhou, 2021 | 1 | 0 | 0 | 1 | 1 | 1 | 1 | 0 | **5** |
| Refaat, 2021 | 0 | 1 | 1 | 0 | 2 | 1 | 0 | 0 | **5** |
| Ahn, 2020 | 1 | 1 | 1 | 1 | 2 | 1 | 0 | 0 | **7** |
| Nam, 2020 | 1 | 1 | 1 | 1 | 2 | 1 | 1 | 0 | **8** |
| Wang, 2020 | 1 | 0 | 1 | 1 | 2 | 0 | 0 | 0 | **5** |
| Won, 2020 | 0 | 1 | 1 | 1 | 2 | 1 | 0 | 0 | **6** |
| Ma, 2020 | 1 | 1 | 1 | 1 | 2 | 1 | 0 | 1 | **8** |
| Qin, 2020 | 1 | 1 | 1 | 1 | 2 | 1 | 1 | 0 | **8** |
| Wu, 2018 | 1 | 0 | 1 | 0 | 2 | 1 | 1 | 0 | **6** |
| Cai, 2017 | 1 | 0 | 0 | 1 | 0 | 1 | 1 | 0 | **4** |
| Ni, 2017 | 0 | 1 | 1 | 0 | 2 | 1 | 1 | 1 | **7** |
| Anggoro, 2016 | 1 | 1 | 1 | 1 | 0 | 1 | 0 | 1 | **6** |
| Onat, 2010 | 0 | 1 | 1 | 1 | 1 | 1 | 1 | 1 | **7** |


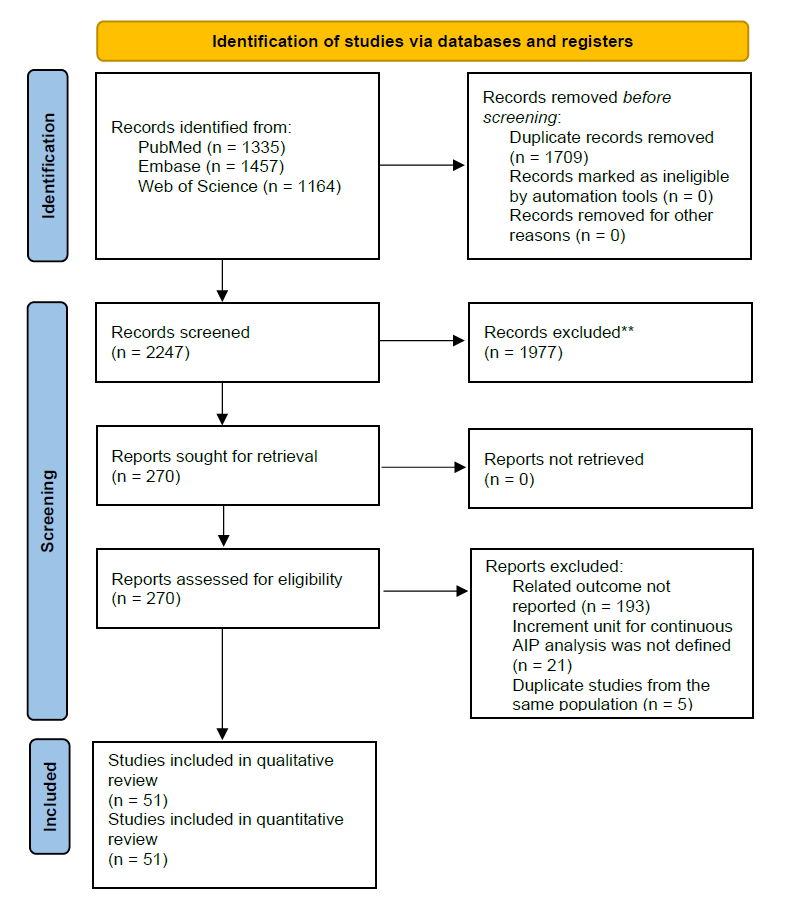


**eFigure 1. PRISMA 2020 flow diagram**


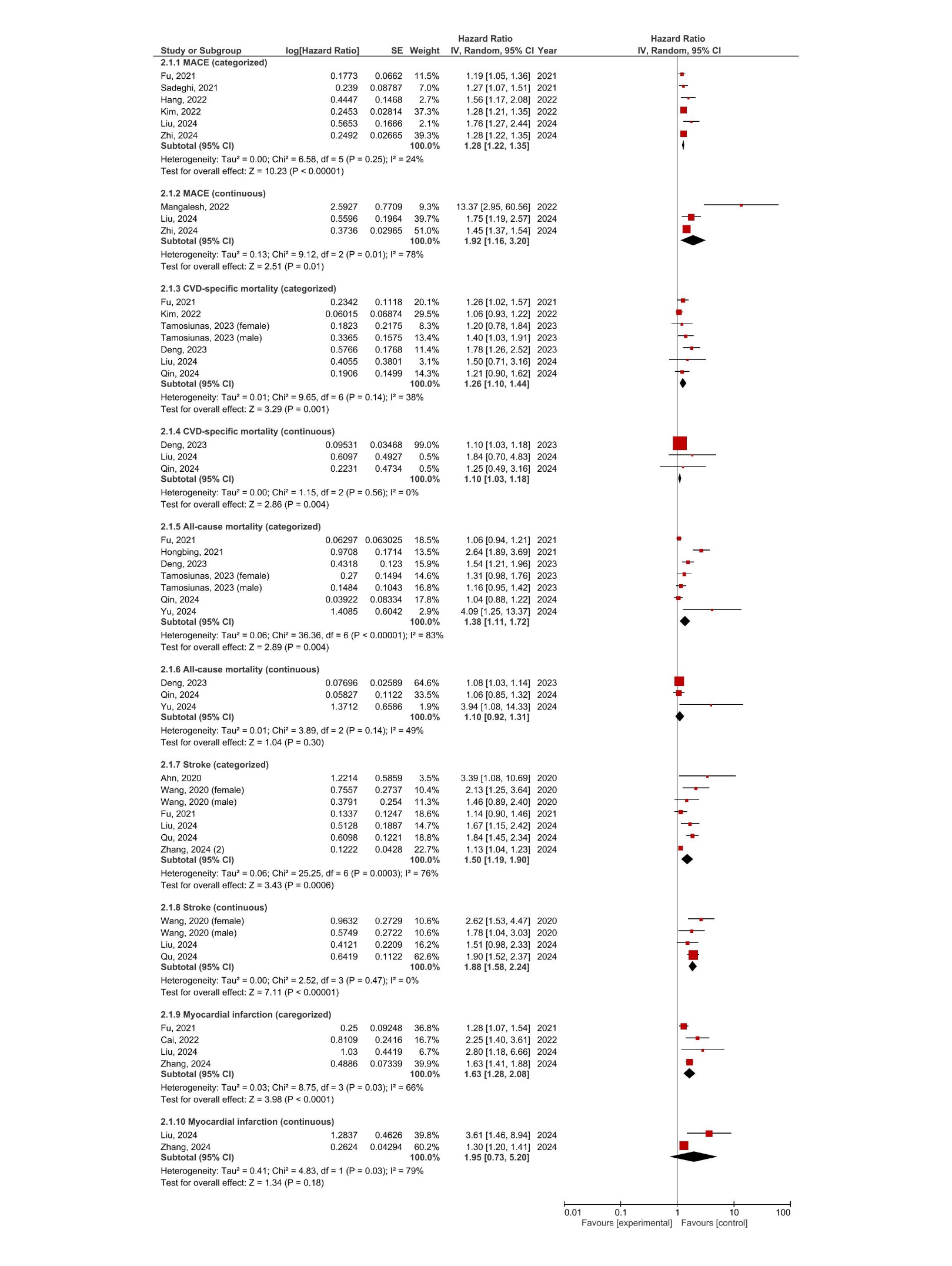


**eFigure 2. Adverse events in populations without established CAD**

**eFigure 3. CAD severity in populations with established CAD**


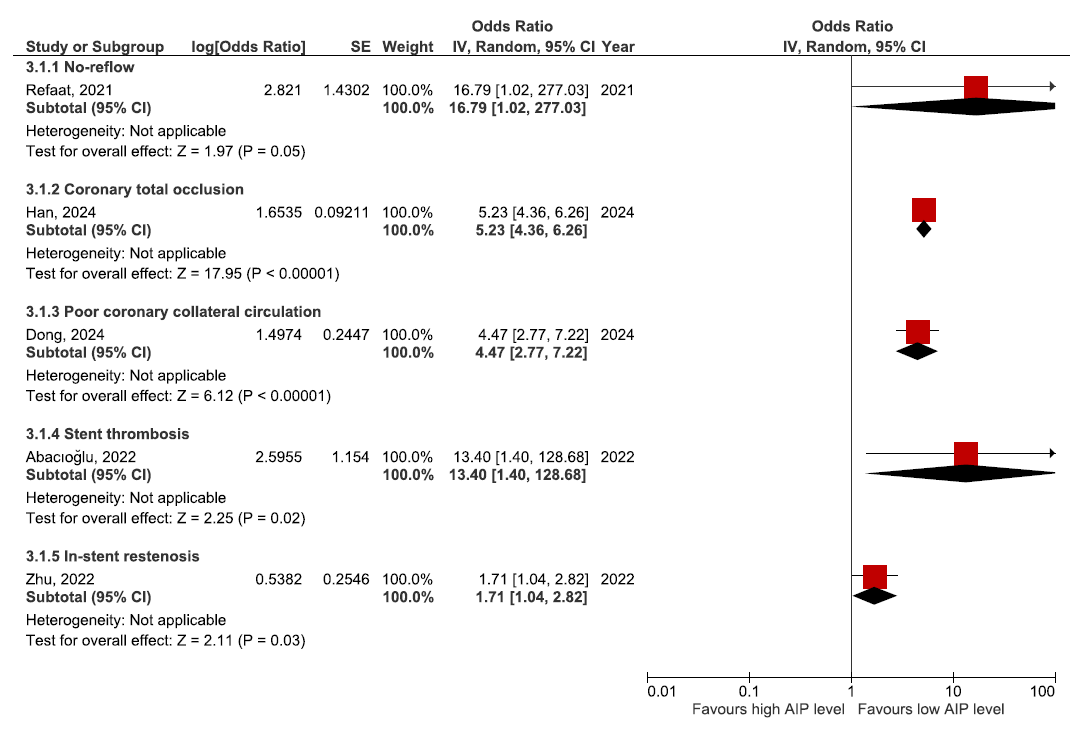


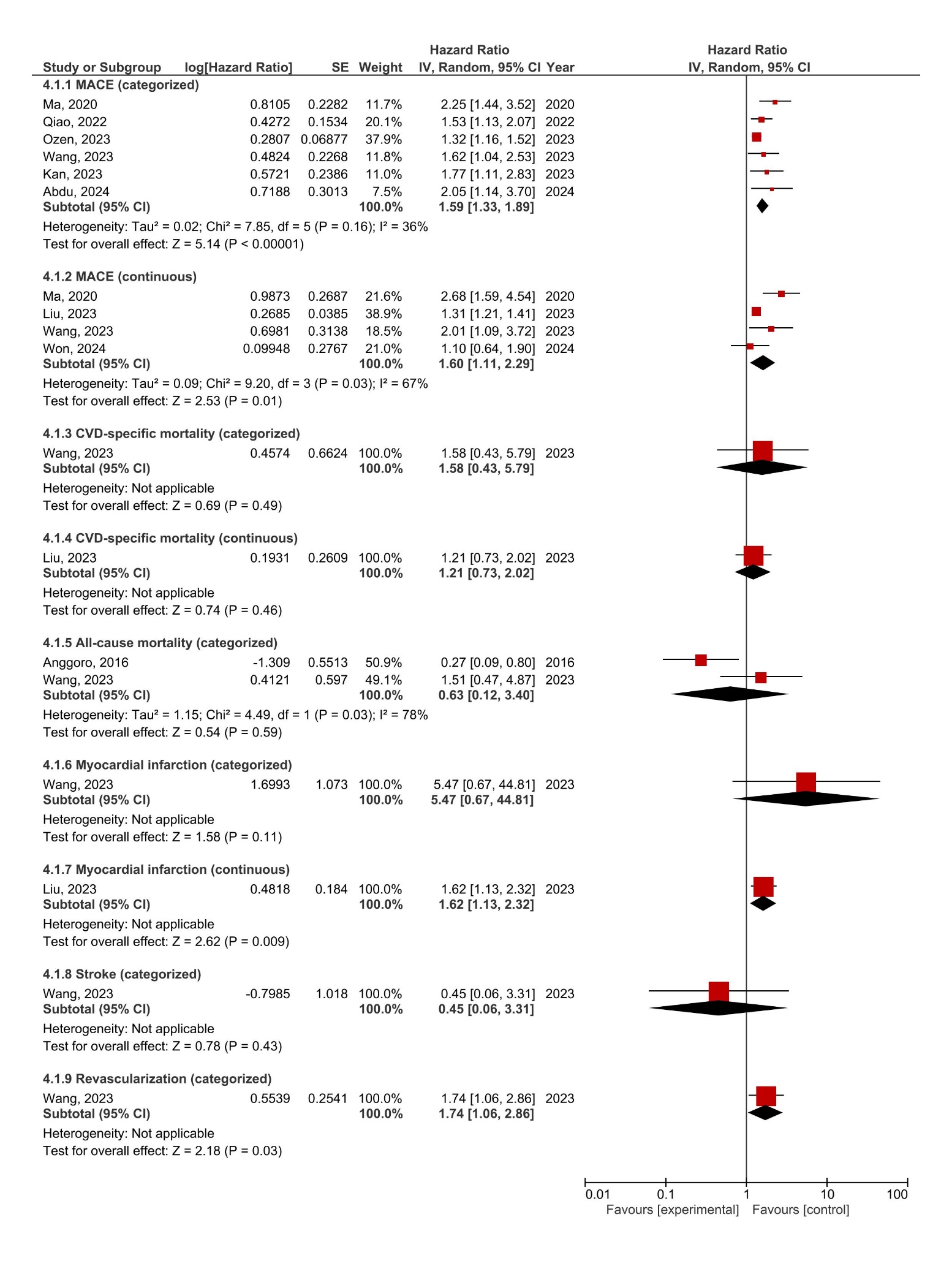


**eFigure 4.** **Adverse events in patients with ACS**


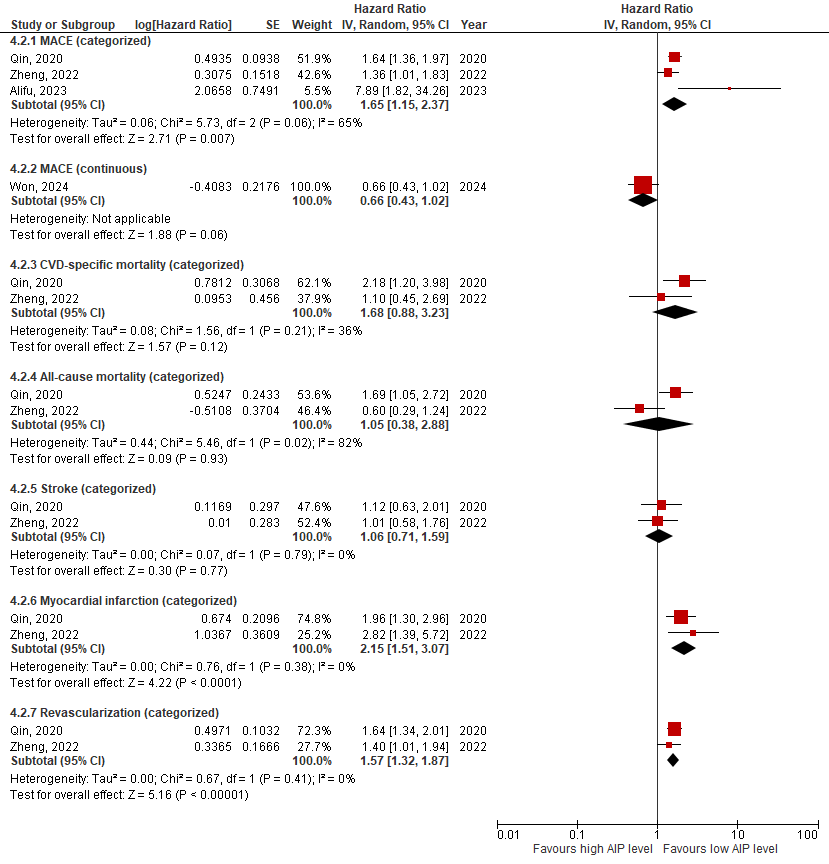
**eFigure 5. Adverse events in patients with CCS or stable CAD**


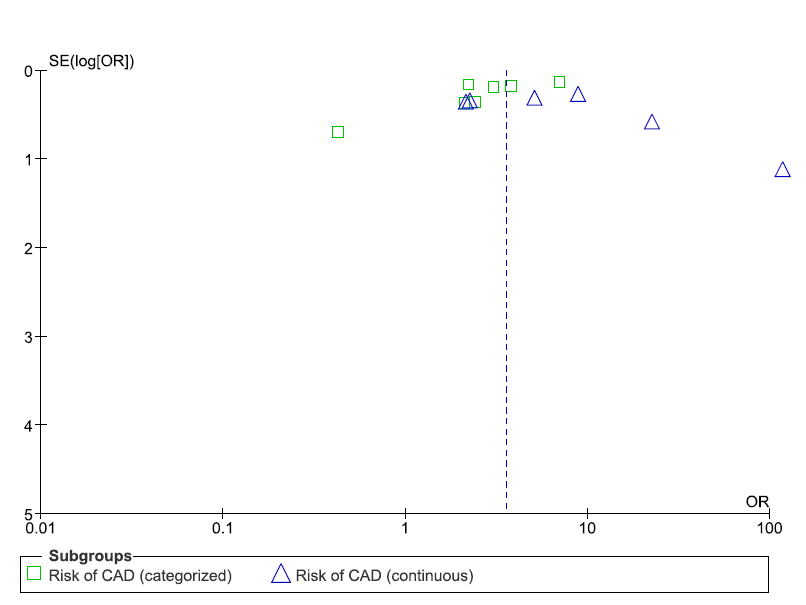


**eFigure 6.** **Funnel plot for risk of CAD**

**eTable 4. Subgroup analyses**

| Outcome | Subgroup | | Studies | OR (95% CI) | Pooled results | Test for subgroup differences |
| --- | --- | --- | --- | --- | --- | --- |
| Risk of CAD (categorized) | Diabetes | Yes | Hu, 2023 | 3.05 (2.07, 4.51) | 2.56 (1.88, 3.49)  I^2^ = 33% | P-value = 0.07 |
|  |  |  | Zhou, 2021 | 2.22 (1.59, 3.10) |  |  |
|  |  | No | Mahdaviroshan, 2022 | 4.17 (2.74, 6.35) | 4.17 (2.74, 6.35) |  |
|  | Gender | Male | Hu, 2023 | 7.54 (4.03, 14.10) | 4.04 (1.16, 14.15) I^2^ = 85% | P-value = 0.12 |
|  |  |  | Ni, 2017 | 2.10 (1.00, 4.41) |  |  |
|  |  | Female | Hu, 2023 | 1.66 (0.96, 2.88) | 0.98 (0.27, 3.57) I^2^ = 69% |  |
|  |  |  | Ni, 2017 | 0.43 (0.11, 1.68) |  |  |
| Risk of CAD (continuous) | Diabetes | Yes | Zhou, 2021 | 1803.89 (13.57, 239812.86) | 1803.89 (13.57, 239812.86) | P-value = 0.05 |
|  |  | No | Mahdaviroshan, 2022 | 11.88 (6.50, 21.71) | 11.88 (6.50, 21.71) |  |
|  | Gender | Male | Onat, 2010 | 2.28 (1.17, 4.44) | 2.28 (1.17, 4.44) | P-value = 0.37 |
|  |  | Female | Onat, 2010 | 2.16 (1.05, 4.44) | 13.48 (0.28, 686.50) I^2^ = 91% |  |
|  |  |  | Wu, 2018 | 117.18 (13.11, 1047.29) |  |  |
| All-cause mortality in populations without established CAD (categorized) | Diabetes | Yes | Fu, 2021 | 1.06 (0.94, 1.20) | 1.27 (0.86, 1.87)  I^2^ = 50% | P-value = 0.69 |
|  |  |  | Qin, 2024 | 1.68 (1.05, 2.69) |  |  |
|  |  |  | Yu, 2024 | 2.64 (0.24, 29.04) |  |  |
|  |  | No | Qin, 2024 | 0.91 (0.48, 1.73) | 1.76 (0.37, 8.45)  I^2^ = 73% |  |
|  |  |  | Yu, 2024 | 4.65 (1.00, 21.62) |  |  |
|  | Gender | Male | Qin, 2024 | 1.31 (0.83, 2.07) | 1.27 (0.93, 1.74)  I^2^ = 33% | P-value = 0.63 |
|  |  |  | Tamosiunas, 2023 | 1.16 (0.95, 1.42) |  |  |
|  |  |  | Yu, 2024 | 4.37 (0.94, 20.32) |  |  |
|  |  | Female | Qin, 2024 | 1.94 (0.97, 3.88) | 1.40 (1.08, 1.83)  I^2^ = 0% |  |
|  |  |  | Tamosiunas, 2023 | 1.31 (0.98, 1.75) |  |  |
|  |  |  | Yu, 2024 | 3.13 (0.33, 29.69) |  |  |
| Stroke in populations without established CAD (categorized) | Diabetes | Yes | Fu, 2021 | 1.14 (0.90, 1.44) | 1.34 (0.95, 1.89)  I^2^ = 77% | P-value = 0.21 |
|  |  |  | Qu, 2024 | 4.58 (1.83, 11.46) |  |  |
|  |  |  | Zhang, 2024 (2) | 1.16 (1.01, 1.33) |  |  |
|  |  | No | Qu, 2024 | 0.96 (0.62, 1.49) | 1.06 (0.95, 1.19)  I^2^ = 0% |  |
|  |  |  | Zhang, 2024 (2) | 1.07 (0.95, 1.21) |  |  |
|  | Gender | Male | Qu, 2024 | 1.79 (1.27, 2.52) | 1.43 (1.18, 1.73)  I^2^ = 51% | P-value = 0.08 |
|  |  |  | Wang, 2020 | 1.78 (1.04, 3.05) |  |  |
|  |  |  | Zhang, 2024 (2) | 1.20 (0.93, 1.55) |  |  |
|  |  | Female | Qu, 2024 | 1.88 (1.34, 2.64) | 1.18 (1.09, 1.28)  I^2^ = 88% |  |
|  |  |  | Wang, 2020 | 2.62 (1.53, 4.49) |  |  |
|  |  |  | Zhang, 2024 (2) | 1.13 (1.04, 1.23) |  |  |
| MACE in populations without established CAD (categorized) | Diabetes | Yes | Zhi, 2024 | 1.28 (1.07, 1.54) | 1.28 (1.07, 1.54) | P-value = 0.31 |
|  |  | No | Mangalesh, 2022 | 13.37 (2.95, 60.59) | 3.90 (0.46, 32.89)  I^2^ = 81% |  |
|  |  |  | Zhi, 2024 | 1.49 (1.41, 1.59) |  |  |
|  | Gender | Male | Zhi, 2024 | 1.39 (1.29, 1.49) | 1.39 (1.29, 1.49) | P-value = 0.07 |
|  |  | Female | Zhi, 2024 | 1.57 (1.41, 1.74) | 1.57 (1.41, 1.74) |  |
| MI in populations without established CAD (continuous) | Gender | Male | Zhang, 2024 | 2.40 (1.40, 4.11) | 2.40 (1.40, 4.11) | P-value = 0.12 |
|  |  | Female | Zhang, 2024 | 1.54 (1.33, 1.78) | 1.54 (1.33, 1.78) |  |
| MI in populations without established CAD (categorized) | Gender | Male | Fu, 2021 | 1.23 (1.09, 1.39) | 1.23 (1.09, 1.39) | P-value = 0.02 |
|  |  | Female | Fu, 2021 | 1.57 (1.32, 1.86) | 1.57 (1.32, 1.86) |  |
| MACE in patients with ACS (continuous) | Diabetes | Yes | Ma, 2020 | 2.68 (1.59, 4.52) | 2.68 (1.59, 4.52) | P-value = 0.008 |
|  |  | No | Liu, 2023 | 1.31 (1.21, 1.42) | 1.31 (1.21, 1.42) |  |
|  | Gender | Male | Ma, 2020 | 2.54 (1.44, 4.48) | 2.54 (1.44, 4.48) | P-value = 0.23 |
|  |  | Female | Ma, 2020 | 5.22 (1.85, 14.75) | 5.22 (1.85, 14.75) |  |
| MACE in patients with CCS (continuous) | Diabetes | Yes | Alifu, 2023 | 0.94 (0.34, 2.63) | 1.58 (1.21, 2.06)  I^2^ = 7% | P-value = 0.64 |
|  |  |  | Qin, 2020 | 1.64 (1.36, 1.98) |  |  |
|  |  | No | Alifu, 2023 | 3.73 (1.40, 9.97) | 2.01 (0.77, 5.27)  I^2^ = 73% |  |
|  |  |  | Zheng, 2022 | 1.36 (1.01, 1.83) |  |  |
|  | Gender | Male | Alifu, 2023 | 2.15 (0.93, 4.97) | 1.43 (1.10, 1.87)  I^2^ = 0% | P-value = 0.61 |
|  |  |  | Zheng, 2022 | 1.37 (1.04, 1.80) |  |  |
|  |  | Female | Alifu, 2023 | 1.77 (0.57, 5.49) | 1.68 (0.98, 2.86)  I^2^ = 0% |  |
|  |  |  | Zheng, 2022 | 1.65 (0.90, 3.03) |  |  |
| All-cause mortality in patients with CCS (categorized) | Diabetes | Yes | Qin, 2020 | 1.69 (1.05, 2.72) | 1.69 (1.05, 2.72) | P-value = 0.02 |
|  |  | No | Zheng, 2022 | 0.60 (0.29, 1.24) | 0.60 (0.29, 1.24) |  |

**eTable 5. Sensitivity analyses**

|  | OR/HR (95% CI) | I^2^ |
| --- | --- | --- |
| **Risk of sub-clinical CAD (categorized)** |  |  |
| Omitting Won, 2020 | 2.53 (1.25, 5.11) | - |
| Omitting Si, 2021 | 3.14 (2.35, 4.20) | - |
| **Risk of CAD (categorized)** |  |  |
| Omitting Liu, 2021 | 2.49 (1.78, 3.49) | 59% |
| Omitting Mahdaviroshan, 2022 | 2.56 (1.44, 4.55) | 88% |
| Omitting Hu, 2023 | 2.68 (1.52, 4.72) | 88% |
| Omitting Wu, 2024 | 2.84 (1.70, 4.74) | 88% |
| Omitting Ni, 2017 (male) | 2.90 (1.75, 4.82) | 87% |
| Omitting Zhou, 2021 | 2.92 (1.74, 4.89) | 84% |
| Omitting Ni, 2017 (female) | 3.24 (2.09, 5.02) | 85% |
| **Risk of CAD (continuous)** |  |  |
| Omitting Wu, 2018 | 5.98 (2.72, 1315) | 82% |
| Omitting Wang, 2021 | 6.45 (2.72, 15.33) | 82% |
| Omitting Zhou, 2021 | 6.74 (3.06, 14.83) | 83% |
| Omitting Mahdaviroshan, 2022 | 8.41 (2.99, 23.60) | 83% |
| Omitting Cai, 2017 | 9.78 (3.35, 28.57) | 86% |
| Omitting Onat, 2010 (male) | 10.61 (4.17, 26.99) | 81% |
| Omitting Onat, 2010 (female) | 10.62 (4.21, 26.79) | 82% |
| **Coronary artery plaque progress (categorized)** |  |  |
| Omitting Shui, 2022 | 1.36 (1.02, 1.82) | 0% |
| Omitting Won, 2021 | 1.49 (0.96, 2.31) | 48% |
| Omitting Nam, 2020 | 1.66 (1.23, 2.26) | 0% |
| **Multivessel lesion (categorized)** |  |  |
| Omitting Hu, 2023 | 1.92 (1.00, 3.71) | - |
| Omitting Wu, 2024 | 2.07 (1.47, 2.94) | - |
| **MACE in populations without established CAD (categorized)** |  |  |
| Omitting Liu, 2024 | 1.28 (1.23, 1.32) | 0% |
| Omitting Hang, 2022 | 1.28 (1.22, 1.33) | 16% |
| Omitting Sadeghi, 2021 | 1.29 (1.22, 1.37) | 39% |
| Omitting Fu, 2021 | 1.30 (1.23, 1.37) | 25% |
| Omitting Zhi, 2024 | 1.30 (1.19, 1.42) | 39% |
| Omitting Kim, 2022 | 1.30 (1.20, 1.42) | 39% |
| **MACE in populations without established CAD (continuous)** |  |  |
| Omitting Mangalesh, 2022 | 1.46 (1.38, 1.55) | 0% |
| Omitting Liu, 2024 | 3.86 (0.45, 33.39) | 68% |
| Omitting Zhi, 2024 | 4.22 (0.59, 30.38) | 85% |
| **CVD-specific mortality in populations without established CAD (categorized)** |  |  |
| Omitting Deng, 2023 | 1.16 (1.05, 1.28) | 0% |
| Omitting Tamosiunas, 2023 (male) | 1.24 (1.07, 1.45) | 42% |
| Omitting Liu, 2024 | 1.26 (1.09, 1.45) | 46% |
| Omitting Tamosiunas, 2023 (female) | 1.28 (1.09, 1.49) | 48% |
| Omitting Fu, 2021 | 1.28 (1.07, 1.52) | 47% |
| Omitting Qin, 2024 | 1.28 (1.09, 1.51) | 46% |
| Omitting Kim, 2022 | 1.34 (1.18, 1.53) | 0% |
| **CVD-specific mortality in populations without established CAD (continuous)** |  |  |
| Omitting Liu, 2024 | 1.10 (1.03, 1.18) | 0% |
| Omitting Qin, 2024 | 1.12 (0.92, 1.38) | 8% |
| Omitting Deng, 2023 | 1.50 (0.77, 2.94) | 0% |
| **All-cause mortality in populations without established CAD (categorized)** |  |  |
| Omitting Qu, 2024 | 1.39 (1.12, 1.73) | 62% |
| Omitting Wang, 2020 (female) | 1.44 (1.13, 1.82) | 76% |
| Omitting Ahn, 2020 | 1.45 (1.16, 1.83) | 77% |
| Omitting Liu, 2024 | 1.48 (1.14, 1.91) | 78% |
| Omitting Wang, 2020 (male) | 1.52 (1.17, 1.96) | 80% |
| Omitting Zhang, 2024 (2) | 1.62 (1.27, 2.06) | 56% |
| Omitting Fu, 2021 | 1.63 (1.21, 2.19) | 80% |
| **All-cause mortality in populations without established CAD (continuous)** |  |  |
| Omitting Yu, 2024 | 1.08 (1.03, 1.13) | 0% |
| Omitting Deng, 2023 | 1.74 (0.50, 6.06) | 74% |
| Omitting Qin, 2024 | 1.75 (0.51, 5.94) | 74% |
| **Stroke in patients without established CAD (categorized)** |  |  |
| Omitting Qu, 2024 | 1.39 (1.12, 1.73) | 62% |
| Omitting Wang, 2020 (female) | 1.44 (1.13, 1.82) | 76% |
| Omitting Ahn, 2020 | 1.45 (1.16, 1.83) | 77% |
| Omitting Liu, 2024 | 1.48 (1.14, 1.91) | 78% |
| Omitting Wang, 2020 (male) | 1.52 (1.17, 1.96) | 80% |
| Omitting Zhang, 2024 (2) | 1.62 (1.27, 2.06) | 56% |
| Omitting Fu, 2021 | 1.63 (1.21, 2.19) | 80% |
| **Stroke in patients without established CAD (continuous)** |  |  |
| Omitting Wang, 2020 (female) | 1.81 (1.50, 2.17) | 0% |
| Omitting Qu, 2024 | 1.86 (1.35, 2.56) | 20% |
| Omitting Wang, 2020 (male) | 1.90 (1.51, 2.38) | 19% |
| Omitting Liu, 2024 | 1.96 (1.62, 2.37) | 0% |
| **Myocardial infarction in populations without established CAD (categorized)** |  |  |
| Omitting Cai, 2022 | 1.52 (1.19, 1.95) | 67% |
| Omitting Liu, 2024 | 1.57 (1.23, 1.99) | 71% |
| Omitting Zhang, 2024 | 1.82 (1.10, 3.00) | 72% |
| Omitting Fu, 2021 | 1.85 (1.41, 2.43) | 32% |
| **Myocardial infarction in populations without established CAD (continuous)** |  |  |
| Omitting Liu, 2024 | 1.30 (1.20, 1.41) | - |
| Omitting Zhang, 2024 | 3.61 (1.46, 8.94) | - |
| **MACE in ACS patients (categorized)** |  |  |
| Omitting Ma, 2020 | 1.42 (1.26, 1.58) | 0% |
| Omitting Abdu, 2024 | 1.55 (1.29, 1.86) | 38% |
| Omitting Kan, 2023 | 1.58 (1.30, 1.93) | 44% |
| Omitting Wang, 2023 | 1.61 (1.31, 1.98) | 47% |
| Omitting Qiao, 2022 | 1.65 (1.31, 2.09) | 48% |
| Omitting Ozen, 2023 | 1.74 (1.45, 2.10) | 0% |
| **MACE in ACS patients (continuous)** |  |  |
| Omitting Ma, 2020 | 1.32 (1.14, 1.54) | 11% |
| Omitting Wang, 2023 | 1.52 (0.99, 2.34) | 73% |
| Omitting Liu, 2023 | 1.81 (1.06, 3.09) | 63% |
| Omitting Won, 2024 | 1.81 (1.10, 2.98) | 77% |
| **All-cause mortality in ACS patients (categorized)** |  |  |
| Omitting Wang, 2023 | 0.27(0.09, 0.80) | - |
| Omitting Anggoro, 2016 | 1.51 (0.47, 4.87) | - |
| **MACE in CCS or stable CAD patients (categorized)** |  |  |
| Omitting Alifu, 2023 | 1.55 (1.31, 1.83) | 8% |
| Omitting Qin, 2020 | 2.81 (0.52, 15.34) | 81% |
| Omitting Zheng, 2022 | 3.02 (0.67, 13.54) | 77% |
| **CVD-specific mortality in CCS or stable CAD patients (categorized)** |  |  |
| Omitting Qin, 2020 | 1.10 (0.45, 2.69) | - |
| Omitting Zheng, 2022 | 2.18 (1.20, 3.98) | - |
| **All-cause mortality in CCS or stable CAD patients (categorized)** |  |  |
| Omitting Qin, 2020 | 0.60 (0.29, 1.24) | - |
| Omitting Zheng, 2022 | 1.69 (1.05, 2.72) | - |
| **Stroke in CCS or stable CAD patients (categorized)** |  |  |
| Omitting Qin, 2020 | 1.01 (0.58, 1.76) | - |
| Omitting Zheng, 2022 | 1.12 (0.63, 2.01) | - |
| **Myocardial infarction in CCS or stable CAD patients (categorized)** |  |  |
| Omitting Zheng, 2022 | 1.96 (1.30, 2.96) | - |
| Omitting Qin, 2020 | 2.82 (1.39, 5.72) | - |
| **Revascularization in CCS or stable CAD patients (categorized)** |  |  |
| Omitting Qin, 2020 | 1.40 (1.01, 1.94) | - |
| Omitting Zheng, 2022 | 1.64 (1.34, 2.01) | - |


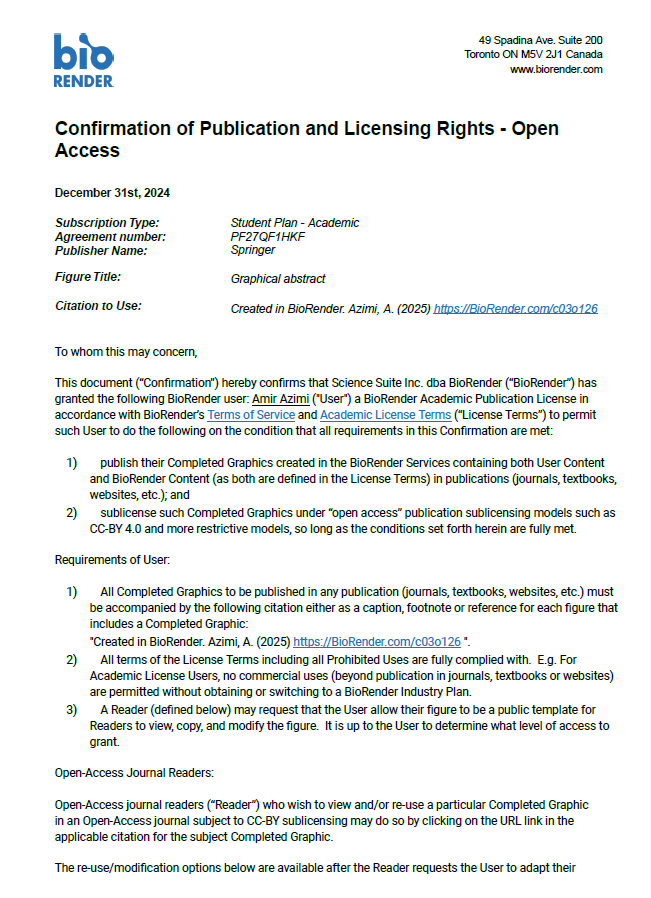


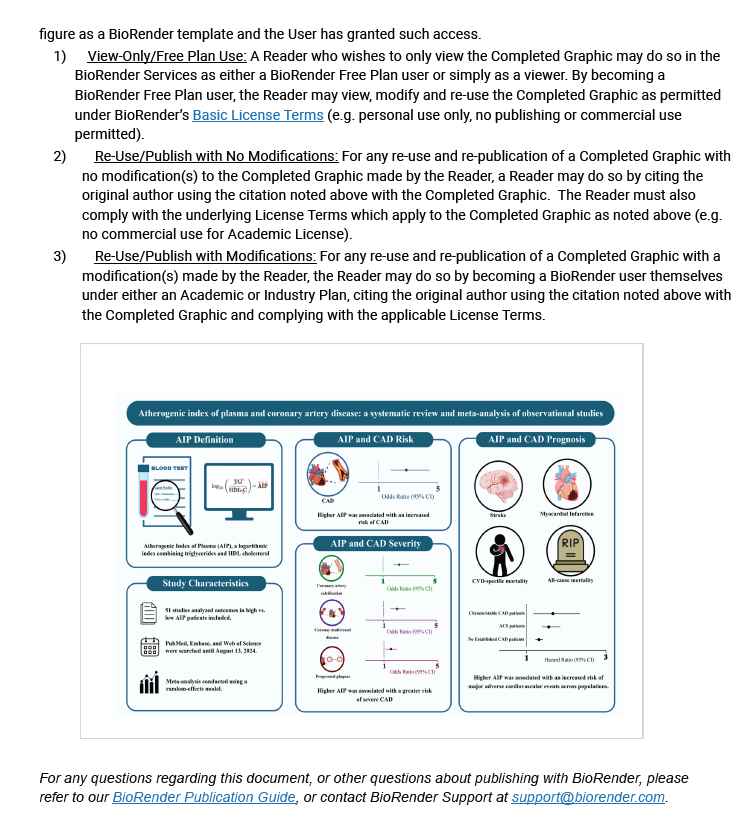

Supplement: Supplementary file 1 — Supplementary Material 1 [file 12933_2025_2582_MOESM1_ESM.docx]
